# Independently Controlled Wing Stroke Patterns in the Fruit Fly *Drosophila melanogaster*

**DOI:** 10.1371/journal.pone.0116813

**Published:** 2015-02-24

**Authors:** Soma Chakraborty, Jan Bartussek, Steven N. Fry, Martin Zapotocky

**Affiliations:** 1 Institute of Physiology, Academy of Sciences of Czech Republic, Videnska 1083, 14220, Prague, Czech Republic; 2 Institute of Biophysics and Informatics, First Faculty of Medicine, Charles University in Prague, Salmovska 1, 12000, Prague, Czech Republic; 3 SciTrackS llc, Lohzelgstrasse 7, CH-8118, Pfaffhausen, Switzerland; University of Arizona, UNITED STATES

## Abstract

Flies achieve supreme flight maneuverability through a small set of miniscule steering muscles attached to the wing base. The fast flight maneuvers arise from precisely timed activation of the steering muscles and the resulting subtle modulation of the wing stroke. In addition, slower modulation of wing kinematics arises from changes in the activity of indirect flight muscles in the thorax. We investigated if these modulations can be described as a superposition of a limited number of elementary deformations of the wing stroke that are under independent physiological control. Using a high-speed computer vision system, we recorded the wing motion of tethered flying fruit flies for up to 12 000 consecutive wing strokes at a sampling rate of 6250 Hz. We then decomposed the joint motion pattern of both wings into components that had the minimal mutual information (a measure of statistical dependence). In 100 flight segments measured from 10 individual flies, we identified 7 distinct types of frequently occurring least-dependent components, each defining a kinematic pattern (a specific deformation of the wing stroke and the sequence of its activation from cycle to cycle). Two of these stroke deformations can be associated with the control of yaw torque and total flight force, respectively. A third deformation involves a change in the downstroke-to-upstroke duration ratio, which is expected to alter the pitch torque. A fourth kinematic pattern consists in the alteration of stroke amplitude with a period of 2 wingbeat cycles, extending for dozens of cycles. Our analysis indicates that these four elementary kinematic patterns can be activated mutually independently, and occur both in isolation and in linear superposition. The results strengthen the available evidence for independent control of yaw torque, pitch torque, and total flight force. Our computational method facilitates systematic identification of novel patterns in large kinematic datasets.

## Introduction

Insect flight provides a powerful model system for neuromotor control [1–3]. Flight puts extreme physiological demands on the organism, which are met by specialized adaptations with sharply defined structure-function relations [4]. This is particularly apparent in flies, in which the generation of power for wing motion and the control of fast modulations of the wing stroke are mediated by two distinct types of muscles [5,6]. In recent years, flight control in flies has been extensively investigated using integrative approaches. In particular, the quantitative correspondence between the kinematic patterns of wing motion and the resulting aerodynamic forces has been clearly established using dynamically scaled robotic models [7–9]. This knowledge provides a functional interpretion of observed variations in the fly’s wing kinematics. Detailed measurement and analysis of wing kinematics therefore has the potential to reveal the functional organization of the flight control apparatus. Our approach combines high-speed measurements of wing motion during extended intervals of unstimulated tethered flight with a computational analysis that extracts independently occurring components of the kinematics.

Freely flying flies execute a variety of flight maneuvers, such as turning, acceleration, and casting flight [10]. To control the aerodynamic forces and torques necessary for the maneuvers, flies modulate their wing kinematics in specific ways [11–15]. For example, yaw torque is known to be generated by bilaterally asymmetric changes in stroke plane angle and the mid-stroke angle of attack [13,14], while a symmetric change in the mean wing translational velocity or in the timing of wing rotation alters the lift force [7,12]. A change in pitch torque can be achieved by a bilaterally symmetric change in mean stroke position [11,15] or in the ratio of down- and upstroke duration [9]. Most of the flight maneuvers require a combination of such changes in wing kinematics. Geurten et al. [16] classified 9 prototypical movements of the hoverfly body, such that any segment of free flight consists of a sequence of these typical movements. 8 out of the 9 prototypical movements involve rotation and/or translation with respect to multiple body axes. A prominent example of flight maneuver is the body saccade, a rapid turn during which the fly changes its heading by up to 120° in about a dozen wingbeat cycles [14]. In the course of the saccade, the fly rotates about all the three body axes; saccades with different combinations of yaw, roll and pitch velocities are observed [10,13,14].

The remarkable maneuverability of flies is based on a special organization of the flight apparatus that permits precise control on very fast time scales. In Dipterans, wing motion is powered by asynchronous flight muscles that act indirectly, by deforming the thorax. Cycle-to-cycle modulations of the wing stroke result from the activity of multiple miniscule steering muscles that are attached to the hinge of each wing [5,6]. This knowledge is based on simultaneous recordings of wing motion and steering muscle activity in the so called „tethered flight”setup (in free flight, electromyographic recording from flight muscles has so far been achieved only in larger insects, such as hawkmoths [17,18]). During tethered flight, the motion of the wings is unconstrained, while the body of the fly is fixated. The observed changes in wing kinematics are interpreted as attempted flight maneuvers or responses. Electrophysiological recordings from groups of steering muscles during tethered flight permitted to link some flight maneuvers to the activity of specific muscles [19,20]. It is currently not fully known, however, which muscles (or synergies of muscles) can be activated independently of each other. In this study, we aimed to identify the kinematic outputs of such independent neuromotor controls. The kinematic output of a given neuromotor control consists of a specific deviation from the baseline wing stroke; such a deviation will be termed an „elementary kinematic pattern“. Our computational analysis operates under the assumption that when multiple independent controls are active simultaneously, the corresponding elementary kinematic patterns superpose linearly to produce the full wing motion. Our results give indirect support for this assumption, but its direct testing is beyond the framework of this article.

To extract the elementary kinematic patterns, we measured and analyzed segments (up to 12 000 wingbeat cycles) of left and right wing motion sampled at 6250 Hz. The tethered flies were not stimulated, but displayed a wide range of kinematic changes during the recordings. These extensive recordings permitted us to apply, for the first time, an advanced statistical analysis designed to identify the full repertoire of independently controlled kinematic patterns. The statistical analysis is based on the method of least-dependent component analysis (LCA) [21]. This is a variant of the well-known independent component analysis (ICA) [22]. In ICA, the set of measured signals is assumed to be a linear combination of statistically independent source signals; in LCA, the assumption of complete statistical independence is relaxed. We allowed a deviation from full statistical independence, as weak dependencies among the elementary kinematic patterns could arise due, e.g., to mechanical coupling through the exoskeleton. We identified the least-dependent components (LDCs) using the MILCA algorithm [21]. MILCA (Mutual Information based Least-dependent Component Analysis) iteratively searches for the set of linear combinations of the measured signals that has the minimal mutual information (which takes into account both linear and nonlinear statistical dependencies). Each LDC specifies the activation time course of a specific deformation mode of the wing stroke. The least-dependent components define the candidates for elementary kinematic patterns—i.e., kinematic patterns that arise from mutually independent neuromotor controls. For clarity we stress that the „stroke deformation modes”in this study do not refer to mechanical deformations of the wing (which are known to be functionally important at least in some larger flying insects [23,24]).

Our analysis starts from just two measured kinematic degrees of freedom—the stroke positions of the left and right wing. To define an input signal for LCA, we extracted the wing stroke position at a given phase of each wing stroke cycle in a given flight recording. LCA based on a set of 16 such signals examines the variability of entire wing strokes cycles, rather than only of pre-selected kinematic parameters such as stroke amplitude. When computing these components, we allowed linear combinations of signals from both wings, which permits to construct, e.g., bilaterally symmetric or antisymmetric deformations of the wing stroke. Table 1 summarizes the nomenclature used throughout the paper.

**Table 1:** Summary of terms and quantities used in the computational analysis.

Term	Definition
Least-dependent component analysis (LCA)	Linear transformation of a set of signals into another set of signals that have minimum mutual dependence.
Stroke trajectory	Time course of stroke positions of left and right wing, as recorded with our apparatus.
Wingbeat cycle (WBC)	Wing motion during the time interval between two consecutive dorsal reversals of the left wing.
Input signal	Stroke position at a specific phase of the wingbeat cycle, over 2500 cycles (see Fig. 2A). 16 such signals (8 from each wing) constitute the full set of input signals to LCA.
Least-dependent component (LDC) or component	A specific linear combination of the input signals, obtained from LCA analysis. It defines the time course of activation of a particular “stroke deformation mode”.
Baseline wing stroke	Set of 16 stroke positions obtained by averaging each of the 16 input signals over the 2500 wingbeat cycles in a given flight segment.
Stroke deformation mode	Specific form of deviation of the wing stroke from the baseline wing stroke. Defined by the separating vector of a specific LDC.
Reconstructed wing stroke	Array of phase points computed by back-transforming a subset of least-dependent components. Used for visualization of “stroke deformation modes”.
Least-dependent kinematic pattern or kinematic pattern	Stroke deformation mode and its time course of activation. (Refers jointly to a specific LDC and its separating vector)
Elementary kinematic pattern	Least-dependent kinematic pattern that is claimed to be controlled independently.
Wiener entropy (WE)	A measure of spectral flatness, defined as the ratio of the geometric mean to the arithmetic mean of the power spectrum of a time series. It ranges from 0 for a sinusoidal wave to 1 for white noise.

In the majority of the analyzed flight segments, the MILCA algorithm converged to a set of linear components that had no significant mutual statistical dependence. Under the assumption that the identified kinematic patterns play a fundamental role for flight control, we expected to find similar occurences in independent trials. Indeed, some of the computed patterns occurred repeatedly across distinct flight segments measured from one fly as well as across individual flies. We provide a classification of such kinematic patterns obtained from recordings of 10 flies. Out of the 7 classified types of kinematic patterns, four showed strong evidence for being considered elementary kinematic patterns. This provides a lower bound for the number of independent neuromotor controls of wing motion. Three identified elementary patterns can be associated with yaw control during body saccades, pitch control, and control of flight power, respectively. The fourth elementary kinematic pattern contains sequences of dozens of wingbeat cycles during which the stroke amplitude of both wings is alternatingly increased and decreased in successive wing strokes. Physiologically, this kinematic pattern may correspond to the activation of a specific steering muscle in every other wingbeat cycle. Multiple elementary kinematic patterns can be active simultaneously in a given stroke cycle; our statistical method allowed us to unambiguously decompose the resulting complex wing strokes into elementary wing stroke deformations.

Our results indicate that changes in yaw torque, pitch torque and total flight force can be controlled mutually independently.The computational method described here provides a novel way to analyze extensive flight recordings, taking advantage of the information inherent in spontaneous (unstimulated) variations of the wing stroke kinematics. Our method of analysis may be productively applied also to other movements with quasiperiodic character, such as animal and human gait.

## Experimental Methods

### Flies

The flies used in the experiments were 5 to 10 days old female fruit flies (*D*. *melanogaster* Meigen), obtained from our laboratory stock descending from 200 wild-caught mated flies. The flies were reared following standard breeding procedures (25 females and 10 males, standard nutritive medium, 12:12 hours’ dark/light cycle). Experiments were performed in the first 6 hours of a subjective day.

### Digital wingbeat analyzer

**Fig 1:**
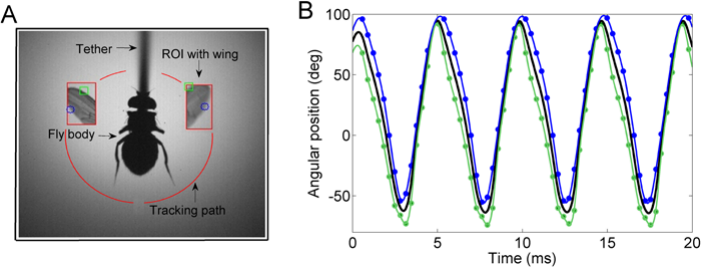
Wing tracking and data post-processing. **A)** Visualization of digital wingbeat analyzer (DWBA) functionality. Two consecutive frames of the dynamically updated region of interest (ROI, left and right wing), are shown overlaid on the mean image of the fly body and the tether. The DWBA detects and records [25] the leading (green squares) and trailing (blue dots) wing edge positions on a predefined tracking path (red circular lines). **B)** Data pre-processing. Recorded positions of the leading and trailing wing edge (green/blue dots) of a single wing are interpolated using B-splines (green/blue trace). The wing chord position (black trace) is obtained as the low-pass filtered mean of the interpolated positions of the two edges. For details see main text.

To measure the wing kinematics of tethered flying flies, we used a computer vision system with real-time analysis functionality (digital wingbeat analyzer—DWBA, SciTracks, Switzerland). The system is based on a high-speed camera (Photonfocus, Switzerland) connected to a frame grabber card in a personal computer. The wings are tracked using dynamic regions of interest (ROI) for increased temporal resolution from localized sampling. An extended Kalman filter fits an *a priori* kinematic model to past wing position measurements, predicting the position of the next ROI. Instead of storing the individual images, the system records the detected positions of the leading and trailing wing edges (Fig. 1A). This procedure greatly reduces the amount of data that is generated and hence allows long recordings of more than 12 000 wingbeats, as required for the statistical analysis.

The angular positions of the leading and trailing wing edges were used to obtain a robust estimate of the wing stroke position (i.e. stroke angle)—see Data preprocessing. Using the DWBA, we achieve a sampling frequency of 3125 Hz for each wing, which at a wingbeat frequency of around 200 Hz corresponds to about 15 stroke position data points per wing stroke (spatial resolution 1°). To avoid tracking a leg instead of a wing edge, the system demands a minimal distance between leading and tailing wing edge of 3 pixels (the width of the legs was usually about 1–2 pixels). For offline verification purposes, the system also saves the last 200 analyzed images. For details on the DWBA functionality see [25].

### Experimental procedure

In each experiment, we cold-anaesthetized a single fly and fixated it by the dorsal part of its thorax to the tip of a steel tether (200 μm diameter, 1 cm length), using UV-hardening glue. The fly was then positioned in the DWBA such that the wing stroke plane agreed with the camera plane as best as possible while avoiding occlusions from the tether (Fig. 1A). Before starting the measurements, the flies were allowed to rest on a piece of wet tissue for at least 30 minutes. We then initiated flight by applying a puff of air on the fly. For each fly, we performed several measurements of 1 minute length, alternating with pauses of 1 minute. The number of recordings per fly varied from 3 to 5, depending on its willingness to fly.

### Data preprocessing

Using a suitable smoothing B-spline interpolation algorithm [26] (Matlab mex implementation by W. Dickson, parameter noise variance set to 3), we up-sampled the leading and trailing wing edge data to a new sampling frequency of 50 000 Hz. We then calculated the center wing position as the low-passed mean of leading and trailing wing edge positions (Matlab zero-phase digital filtering, 3^rd^ order Butterworth filter with cut off frequency 1500 Hz). For an example, refer to Fig. 1B.

When comparing the control images with the wing position data, we found that when both wings came close together during the dorsal reversal, the DWBA occasionally lost track of one of the wings and was tracking an edge of the other wing instead. This type of artifact leads to typical deformations of the normally roughly sinusoidal wing trajectory, comprising a second local maximum close to the dorsal reversal. To exclude intervals that were potentially contaminated by these types of mistrackings, we applied a custom-built Matlab algorithm that automatically discarded wingbeat cycles in which two dorsal maxima occurred within 3 ms. We also discarded the corresponding wingbeat cycles of the other wing and the nearest neighboring cycles.

## Computational Methods

### Signal extraction

**Fig 2:**
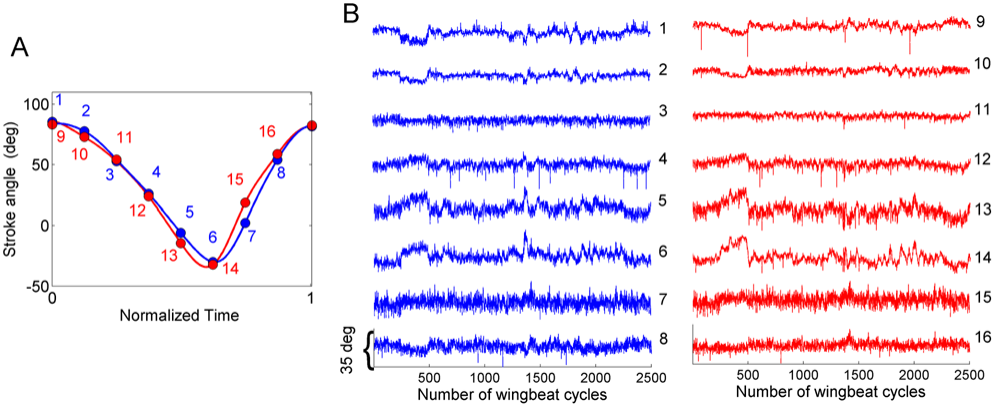
Definition of phase points and an example of their time course. **A)** One cycle of the stroke trajectory of left wing (blue) and right wing (red). The cycle is divided into 8 phase points sampled at equal time intervals; the angular positions of the two wings at these 8 phase points define the 16 signals X_i_. **B)** Time course of the 16 signals X_i_ during a flight segment of 2500 wing stroke cycles. See Fig. 3 for the least dependent components of the signals shown here.

After the preprocessing of experimental data we are left with two time series—the left and right wing stroke trajectories (Fig. 1B). To identify statistically independent wing stroke variations, we first extract a new set of signals, each corresponding to a specific phase of the wingbeat cycle. We define a stroke cycle as the time interval between two consecutive dorsal reversals of the stroke angle of the left wing. From the wing stroke trajectory in each cycle, we extract 8 stroke position values, sampled at 8 temporally equidistant phases of the stroke cycle. An example for one wing stroke is shown in Fig. 2A. Each of the 16 phase points (1 to 8 from the left and 9 to 16 from the right wing) then defines a separate time series, with successive samples of the series corresponding to successive wing strokes. An example of the 16 time series for a flight segment of 2500 cycles (approximately 12–13 seconds) is shown in Fig. 2B. Note that in distinction to the original recorded data (the wing stroke trajectory sampled at 3125 Hz), the 16 time series based on the phase points are not periodic. (A related procedure was used to define the input signals for principal component analysis in recent investigations of human movements [27,28].)

In this manner we obtain a multivariate signal **X** = [*X*_*1*_… *X*_*16*_]^T^ that can be used as the input for least dependent component analysis (see next subsection). We consider only 8 phase points per wing as further increasing this number (within the limit of experimental sampling rate of ~15 per cycle) did not yield new features but considerably increased the computation time. The effect of increasing or decreasing the number of analyzed phase points will be described in the Discussion using a specific example. The choice of the dorsal stroke reversal point as the beginning of wing stroke is arbitrary; we were lead to it by the knowledge that the ventral reversal and mid-stroke phases of the wingbeat are considered as more important for flight control than the dorsal reversal phase [29]. Putting the cycle boundary at the dorsal reversal point results in the inability to construct the wing velocity just before the dorsal reversal, as this would require a linear combination of point 1 from a given cycle with point 8 from the previous cycle, while in our analysis, only linear combinations of phase points from the same cycle are included (see next subsection). At the ventral reversal and mid-stroke phases, no such restriction arises.

It is important to note that the procedure for constructing the 16 signals *X*_*i*_ results in the loss of information about the duration of each cycle. The signals *X*_*i*_ therefore reflect only variations in the waveform (shape) of the wing stroke, but not changes in the stroke duration / wingbeat frequency. In our analysis, we assess separately if the obtained least-dependent components of the stroke waveform are correlated (or uncorrelated) with the stroke period.

For the purpose of LCA analysis, all flight recordings were divided into segments of a fixed length, and the least-dependent components were computed separately for each segment. When choosing the segment length, the following two aspects were considered. Long-duration segments are more likely to contain multiple occurrences of distinct kinematic changes, which is a requirement for successful statistical analysis. On the other hand, for long segments the signal **X** may violate conditions of wide-sense stationarity, a pre-requisite of LCA analysis [22]. Considering this tradeoff, we found the duration of 2500 wingbeat cycles to be approximately optimal. We use this segment length throughout our analysis.

To recapitulate, each sample of the multivariate signal **X** defines the waveform of one stroke cycle, for both the left and right wing. Our analysis exploits the variability of this signal over 2500 successive samples.

### Algorithm for finding least-dependent components of the wing trajectory

To extract the least-dependent component variations of wing trajectory we used the computational tool least-dependent component analysis (LCA). The algorithm searches for the linear transformation of the input signals *X*_*i*_ such that the transformed signals are mutually statistically independent to the largest possible degree. While it is always possible to find a linear transformation resulting in uncorrelated transformed signals (e.g., the Karhunen-Loeve transform in principal component analysis), the LCA algorithm goes further and attempts to achieve full statistical independence. The coefficients of the optimal linear transformation define the separating matrix **W**. The transformed signals *Z*_*i*_, given by

$$Z_{i}=w_{i1}X_{1}+w_{i2}X_{2}+\ldots +w_{i16}X_{16}\text{; i.e. }Z\text{ = }\mathrm{WX},$$ (1)

are the least-dependent components. For each component *Z*_*i*_, the coefficients [*w*_*i1*_…, *w*_*i16*_] define the corresponding separating vector. LCA is a variant of the well-known independent component analysis (ICA) [22]. In the special case when the input signals are a linear combination of fully statistically independent sources, LCA and ICA give identical results, i.e., the least dependent components are then equivalent to independent components.

In LCA analysis a reliable estimator of mutual dependence of variables is crucial, because it is needed as a cost function for optimization. The algorithm we use quantifies dependence by estimating mutual information. Mutual information between two random variables is defined as the reduction in the uncertainty of one due to the knowledge of the other. It is a more complete measure of independence than the well-known Pearson correlation coefficient because it quantifies the entire dependence structure, both its linear and non-linear parts. Two random variables are fully statistically independent if and only if their mutual information is zero. The mutual information of two random variables *Q* and *R* can be calculated as

I(Q,R)=H(Q)+H(R)-H(Q,R), (2)

where H(*Q*) and H(*R*) are the marginal entropies and H(*Q*,*R*) is the joint entropy. (The (differential) entropy of a variable *Q* with probability density p(*Q*) is defined as $\text{H}(Q)=-\int_{-\infty }^{\infty }\text{p}(Q)\text{log}\text{p}(Q)dQ$. To define the joint entropy H(*Q*,*R*) of two variables, the joint probability density p(*Q*,*R*) is used.)

For a multivariate signal like **Z** in our case, the definition in Eq. 2 can be extended to define the joint mutual information of **Z** as:

$$\text{I}(Z_{1},\ldots ,Z_{16})=\sum_{i=1}^{16}\text{H}(Z_{i})-\text{H}(Z_{1},\ldots ,Z_{16}).$$ (3)

Reliable estimation of entropy from a finite number of samples of the random variables is a non-trivial task [30]. When calculating combinations of entropies (such as in Eqs.2, 3) that can have a total value close to zero, it is particularly important to eliminate any biases in entropy estimation. Kraskov et al. developed a reliable mutual information estimator [31] based on a previously known binless strategy for entropy estimation. This estimator is the basis for the MILCA algorithm (mutual information based least dependent component analysis [21]) which we use in this study. In searching for the least dependent components, the MILCA algorithm iteratively remixes the input signals, converging to linear combinations with minimal joint mutual information (Eq. 3). A brief description of this algorithm is provided in the S1 Text; for further details refer to the original article [21]. We used the Matlab implementation of MILCA downloaded from www.ucl.ac.uk/ion/departments/sobell/Research/RLemon/MILCA/MILCA. Parameters were set as follows: distance to the 12^th^ neighbor and rectangular 2D neighborhood for entropy estimation; first two Fourier components in the fitting of mutual information vs. rotation angle curve.

**Fig 3:**
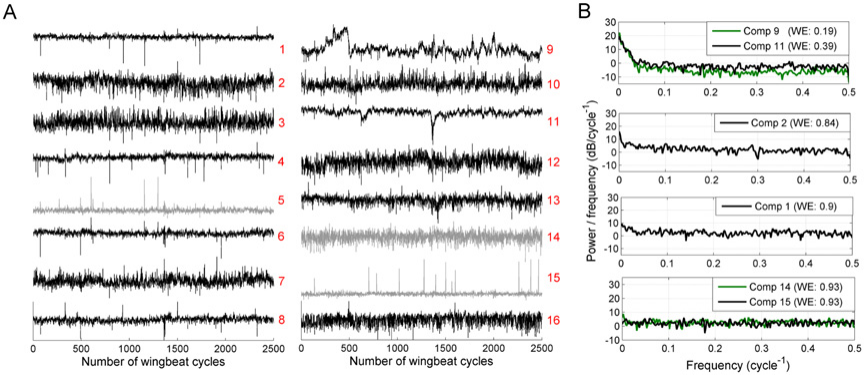
Least-dependent components and their temporal features. **A)** Least-dependent components estimated from the 16 signals in Fig. 2B. Components with noise-like flat power spectra (Wiener entropy > 0.9) are plotted in gray. **B)** Welch power spectra and the Wiener entropy (WE) values of selected components shown in A.

Fig. 3A shows the least-dependent components (set of 16 time series *Z*_*i*_) obtained from LCA analysis of the wing stroke signals (set of 16 time series *X*_*i*_) shown in Fig. 2B. These components will be discussed in detail in Results.

### Least-dependent kinematic pattern

Each least-dependent component *Z*_*i*_ is a series of 2500 sample points, with one sample per stroke cycle. The value of *Z*_*i*_ in a given cycle gives the weight of a particular stroke deformation mode in the overall wing stroke trajectory. This stroke deformation mode is defined by the coefficients of the corresponding separating vector (i.e. i^th^ row of the separating matrix **W**). For some components that we obtain, direct examination of the separating vector is sufficient to understand the corresponding stroke deformation mode. For example, components of type IV (see Results) are obtained as the difference of the wing position signals *X*_*1*_ and *X*_*9*_; the corresponding stroke deformation mode therefore consists of an antisymmetric change of the wing position at dorsal stroke reversal for the two wings. In general, however, it is more convenient to construct a graphical representation of the stroke deformation mode defined by the separating vector of a given component.

To do so, consider the inverse of Eq. 1,

$$X=W^{-1}Z\text{.}$$ (4)

The mixing matrix **W** ^**-1**^ defines the transformation from least-dependent components to original signals. When the full set of components **Z** = [*Z*_*1*_, …, *Z*_*16*_]^T^ is used in Eq. 4, the 16 signals **X** are faithfully reconstructed. Equation 4 can be expanded as

$$X=W^{-1}\langle Z\rangle +W^{-1}(Z-\langle Z\rangle )=\langle X\rangle +W^{-1}\hat{Z},$$ (5)

**Fig 4:**
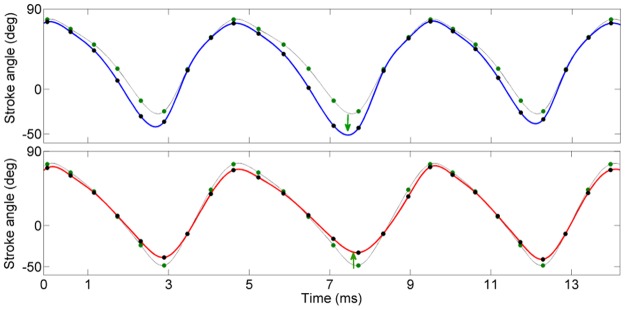
Reconstructed wing stroke, with deviation from baseline stroke due to a selected kinematic pattern. (Upper panel: left wing, lower panel: right wing). The green dots and the interpolated gray lines show the baseline stroke trajectory. The black dots (interpolated with blue and red line) are phase points reconstructed from only one selected component (see main text). The 8 reconstructed phase points for each wing are converted to time points, based on the recorded wing stroke duration. This stroke deformation mode is seen to consist of an increase in left ventral amplitude with simultaneous decrease in right ventral amplitude.

Where 〈.〉 denotes the average over all wing strokes. The vector $\hat{Z}=Z-\langle Z\rangle$ contains the deviations of the weights from their respective mean values. The wing stroke is thus represented as the sum of the baseline wing stroke 〈X〉 (i.e., the mean stroke, averaged over the entire flight segment) and the wing stroke deformation $W^{-1}\hat{Z}$. To construct the stroke deformation mode corresponding purely to the i-th component, we suppress the stroke deformations due to other components—i.e., in the second term of Eq. 5, we replace all coefficients in **W** ^**-1**^, except in its i^th^ column, by zeros. From such partial reconstruction we obtain an array of phase points called the reconstructed stroke cycle, which when plotted together with the baseline wing stroke gives a visualization of the stroke deformation. In Fig. 4, we show an example of three reconstructed stroke cycles, with only one stroke deformation mode included. Comparing this to the baseline wing stroke shown in gray, it is seen that in this example, the stroke deformation mode is mainly an anti-symmetric change in ventral amplitudes (i.e., wing positions at ventral stroke reversal).

Each stroke deformation mode is thus a specific form of deviation from the baseline wing stroke. Deviation of any element of ${\hat{Z}}_{i}$ from zero implies that the stroke deformation mode represented by the i^th^ LDC is active at that stroke cycle. Thus each LDC specifies the time course of activation of the stroke deformation mode it encodes. Together, the activation time course and the stroke deformation mode constitute a least-dependent kinematic pattern.

### Selective removal of measurement errors by LCA-based reconstruction

Reconstruction of the stroke cycle from least-dependent components (Eq. 5) can be used as a systematic method for removing experimental artifacts from the recorded data. In the recordings from our digital wingbeat analyzer, an occasional source of measurement error is the mistracking of the wing edges; wing strokes with mistrackings near the dorsal reversal were removed in the data preprocessing step (see Methods). Mistrackings during other parts of the wing stroke, however, may persist and appear as isolated single-cycle jumps in one or more of the 16 signals.

**Fig 5:**
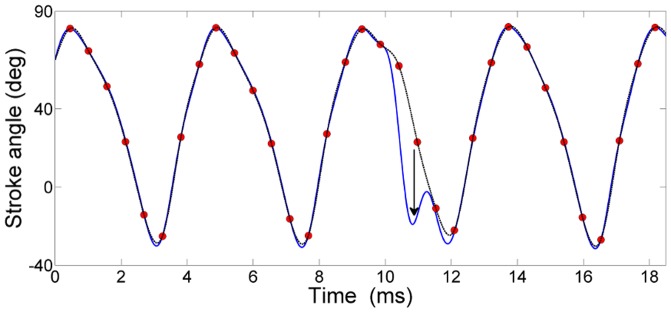
LCA-based denoising of the wing trajectory. Blue: interpolated trajectory of the wing center position as obtained from the wingbeat analyzer. Red circles: phase points obtained by inverse-transforming the set of least-dependent components while omitting a particular noise-like kinematic pattern (component 12 in Fig. 6B). Black dashed line: interpolated denoised stroke trajectory. Black arrow points to the mistracking which has been selectively removed. The 8 reconstructed phase points are converted to time points, based on the recorded wing stroke duration.

**Fig 6:**
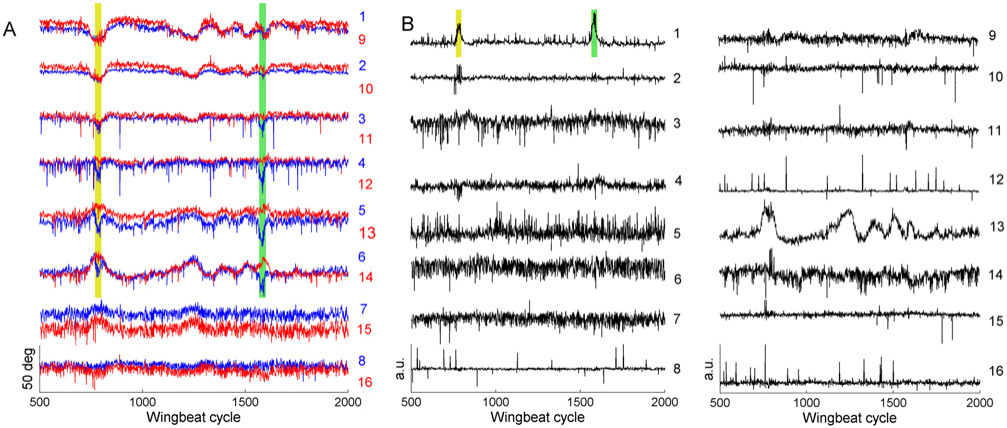
Least-dependent components separate distinct temporal features. **A)** 16 signals extracted from a flight segment of 2500 wingbeat cycles (the signals are numbered according to Fig. 2A). Red: signals from left wing; Blue: signals from right wing. Yellow and green highlighted regions mark two events with pronounced variations asymmetric in the two wings. **B)** Least-dependent components of signals shown in A. Component 12 encodes the kind of mistracking shown in Fig. 5 and the red dot marks the stroke cycle corrected in Fig. 5. Note: the examples in Fig. 6 and in Figs. 2 and 3 are from two different flies.

While it would be possible to use a frequency-domain filter to remove these mistrackings, this would result in removal of all variations with a single-cycle time scale, including true wing motion patterns (type VI kinematic patterns, Sec. Results). In order to selectively remove mistrackings one can exploit the fact that in general, their occurrence is statistically independent from actively controlled wing stroke deformations. Most of the cases of mistrackings therefore appear in a separate LDC. To remove these mistrackings one needs to reconstruct the phase points from the full set of LDCs while suppressing the component(s) encoding mistrackings. Fig. 5 shows an example where a case of mistracking during downstroke (marked by arrow) has been selectively removed in this manner (component 12 encoding this mistracking is shown in Fig. 6B; the cycle with mistracking is marked by a red dot). In this rare case, a moving leg (whose width transiently exceeded 3 pixels) was tracked instead of a wing edge.

The MATLAB code for the computational analysis, as well as the input kinematic data and the obtained least-dependent components, are available through figshare at http://dx.doi.org/10.6084/m9.figshare.1244993.

## Results

We present the results in four subsections. In the first three subsections, we present important general features of the wing motion decomposition into least-dependent kinematic patterns, illustrated with specific examples. The fourth subsection provides a classification of the kinematic patterns obtained frequently across 100 test flights from 10 flies, and discusses the correspondence of some of these patterns to known flight maneuvers.

### Events with distinct time courses are separated into distinct least-dependent components

The time course of each of the 16 input signals *X*_*i*_ contains events with a variety of durations and forms. As an example, Fig. 6A shows the 16 signals over 1500 wingbeat cycles from a typical recording (recall that each signal *X*_*i*_ is the sequence of stroke angles at a fixed phase of the wingbeat cycles—see Table 1). Each event typically appears in multiple input signals. For example, events occurring symmetrically in left and right wings dominate signals 1, 9, 2, 10, 5, 13, 6 and 14. In addition to these events, sharply peaked events anti-symmetric in the two wings are present in signals 5, 13, 6 and 14 (see highlighted regions in Fig. 6A). In signal 4 the latter are the dominant feature.

The least-dependent component analysis separates events of distinct types into distinct components. Fig. 6B shows the LDCs of the signals from Fig. 6A. In this example, the separation of activation events is as follows:

- Events symmetric in the two wings are separated in component 13.
- Events anti-symmetric in left and right wing (the two sharply peaked events highlighted in Fig. 6A) are isolated in component 1.
- Gradual drifts occurring at a timescale of many hundreds of wingbeat cycles are separated in 3^rd^ and 14^th^ components.
- Several components (8, 12, 15, 16) consist of isolated short pulses with a duration 1–2 cycles. Such pulses arise mainly in the input signals *X*_*3*_, *X*_*4*_ and *X*_*11*_, *X*_*12*_ (phases corresponding to mid-stroke) and are due predominantly to mistracking of the wing (tracking the wing vein or leg instead of wing edges—see arrow mark in Fig. 5).

Another example is seen in Fig. 3A. The triangular-shaped event seen early in component 9 is apparent in most of the 16 input signals (Fig. 2B), but is isolated in only one component (Fig. 3A). The two brief events in component 11 do not stand out in the input signals; least-dependent analysis, however, isolates these as events of a particular type. Notice that these two events are similar in time course to the events in component 1 of Fig. 6B (obtained from a flight recording of a different fly). This is an example of repeated occurrence of components; such components will be analyzed in detail in the last subsection.

The separation of temporal features into distinct components, illustrated in the examples given above, is a typical outcome of the LCA analysis. In rare cases, the separation fails, and several components will contain the same event. In general, however, LCA provides a powerful tool for extracting and sorting the various temporal features of the input signals, as well as for isolating experimental artifacts (see Computational Methods).

### The least-dependent components are statistically nearly independent

The main goal of our study was to identify kinematic patterns that are controlled independently of each other (for example, through parallel neural pathways that activate different steering muscles). If two types of kinematic patterns cannot be controlled independently, then the time courses of their activation will necessarily have some degree of statistical dependence—even during spontaneous, unstimulated flight behavior. Consequently, if the components obtained from LCA are fully statistically independent, they define candidates for elementary kinematic patterns.

**Fig 7:**
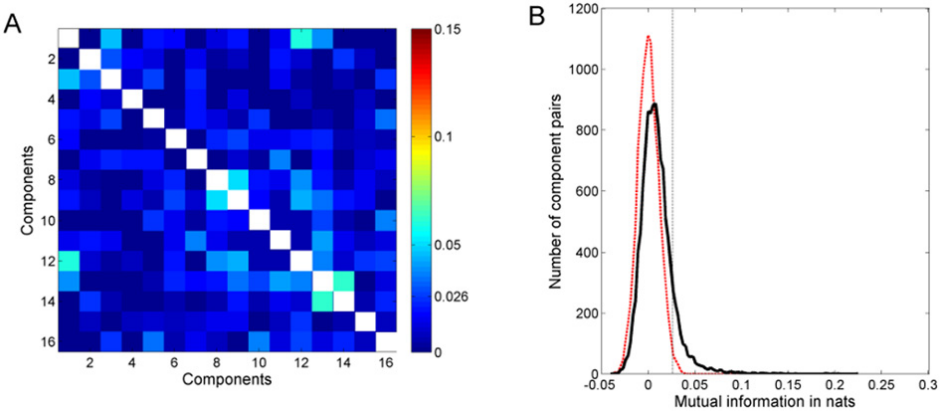
Mutual information of least-dependent components. **A)** Dependency matrix of the components shown in Fig. 6. The color scale indicates the value of mutual information in nats. Values below 0.026 nats imply statistical independence. **B)** Solid black curve shows the distribution of pairwise mutual information, for pairs of components in a flight segment, over a total of 100 flight segments. Dashed red curve gives the null estimate of this distribution, obtained after randomly reshuffling the activation time course of each component. Dashed black line marks the α = 0.01 confidence limit for rejecting the null hypothesis of zero mutual information.

The least-dependent component analysis produces the most independent linear combinations of the input signals. To evaluate if the resulting LDCs are fully statistically independent or not, it is necessary to examine their mutual information. Fig. 7A shows the matrix of estimated pairwise mutual information for the least-dependent components in Fig. 6B. Mutual information was calculated using Eq. 3 and the entropies using the algorithm presented in [31]. (Depending on the base of the logarithm in the definition of entropy, mutual information is expressed either in bits (base 2) or nats (base e). The conversion factor is: 1 nat = (1 / ln 2) bits = 1.44 bits.) To determine the significance of mutual information values obtained from the estimator, the statistics of null estimates of mutual information was obtained. To do so, the time series of each of the 16 LDCs from a given flight segment was randomly reshuffled to destroy any residual dependence with other components and then the pairwise mutual information was estimated. The distribution of this null estimate calculated from 100 flight segments (12000 pairs of LDCs) is shown in Fig. 6B (red dashed line). Based on this distribution (fitted to a Gaussian distribution with zero mean), only values above 0.026 nats were considered as significant (α value of 0.01).

In the example in Fig. 7A, all but 10 of the 120 pairs of components have mutual information less than 0.026 nats and hence are pairwise statistically independent. 9 out of the remaining 10 pairs (1–3, 1–13, 5–16, 7–11, 8–9, 8–12, 9–12, 11–13 and 13–14) have mutual information less than 0.04 nats, which is only nominal statistical dependence. Only one pair (i.e. 1–12) has a significant mutual information of 0.06 nats. To obtain a similar statistics for the entire set of 100 flight segments, pairwise mutual information of LDCs from individual segments were estimated (12000 pairs of LDCs). The solid black line in Fig. 7B shows their distribution. 95.5% of pairs were statistically independent and only 0.5% shared mutual information greater than 0.1 nats, testifying that components with large statistical dependence are rare.

### Statistical analysis allows to decompose complex stroke trajectories into elementary kinematic patterns

**Fig 8:**
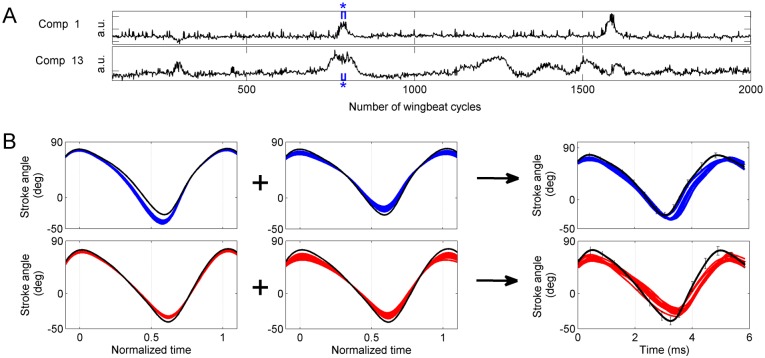
Complex changes in stroke trajectory resolved into elementary kinematic patterns. **A)** Activation time course of two kinematic patterns (components 1 and 13 from Fig. 6B). **B)** *Left panel*: 10 consecutive cycles (marked with brackets and asterisk symbol in A) of wing stroke deformation reconstructed from only component 1. The reconstructed phase points (not shown for clarity) were interpolated with cubic splines. Blue: left wing, red: right wing. The black line shows the baseline wing stroke. *Middle panel*: 10 consecutive cycles (marked with brackets and asterisk symbol in A) of wing stroke deformation reconstructed from only component 13. *Right panel*: Wing stroke trajectories during these 10 cycles. The trajectory deformation in the right panel is resolved into a linear combination of laterally symmetric and anti-symmetric deformations.

As discussed above, events with distinct time courses are typically separated into distinct components. These distinct types of events occasionally overlap in time. An example in seen in components 1 and 13 from Fig. 6B, shown again in Fig. 8A for clarity. Events in these two components have distinct time scales; moreover, events in component 1 are accompanied by correlated changes in wingbeat period, whereas those in component 13 are not (correlation coefficients 0.68 and 0.08, respectively). The time courses of these two components are statistically nearly independent (the mutual information evaluated over the whole flight segment of 2500 wingbeat cycles is 0.04 nats). Some activation events, however, occur simultaneously: the first major event in component 1 overlaps in time with a major event in component 13. The stroke deformation modes encoded by components 1 and 13 are shown in the left and middle panels of Fig. 8B. Component 1 encodes for a change in ventral amplitude that occurs antisymmetrically in the left and right wing, while component 13 encodes a decrease in stroke amplitude occurring symmetrically in both wings.

The right panel of Fig. 8B shows the recorded wing stroke trajectories of 10 wingbeat cycles (marked with blue bracket and asterisk in Fig. 8A), compared to the baseline trajectory (black). The recorded strokes deviate from the baseline stroke by asymmetric changes in ventral and dorsal amplitudes combined with an increase in stroke duration. Based only on this information, it would not be possible to deduce that such a complex deviation is generated by a specific linear combination of more fundamental, independently controlled stroke deformation modes. Based on our statistical analysis, however, we can conclude that the deformation of the stroke trajectory shown in the right panel of Fig. 8B is a composite of the elementary stroke deformation modes shown in the left and middle panels of Fig. 8B. This conclusion is possible only after examining (using LCA) the entire segment of 2500 cycles, in which the elementary stroke deformations are seen to occur independently of each other.

Similar cases of composite kinematic changes that arise as a superposition of several elementary kinematic patterns are encountered also in other examined flight segments. A second example is shown in S1 Fig.

### Classification of frequently obtained least-dependent kinematic patterns

In total, 100 flight segments from 10 flies were analyzed. LCA was carried out in each of these segments, generating a set of 100 x 16 = 1600 least-dependent components, each corresponding to a separating vector. Kinematic patterns with certain features were found repeatedly within this set. These kinematic patterns represent stroke deformation modes exercised frequently by different flies, and can thus be presumed to be important for flight control. To characterize such kinematic patterns we classified them into well-defined types.

We first divided the kinematic patterns into those showing distinct temporal features and those resembling broad-band noise. As an example, consider the set of components shown in Fig. 3A. The activation time course of component 14 appears featureless, while for component 15 it is dominated by randomly occurring single cycle jumps. As seen in Fig. 3B, the Welch power spectra for both these components are flat. We view such components as not relevant for flight control, and drop them from further analysis. To quantify the spectral flatness, we estimated the Wiener entropy (WE), defined as the ratio of the geometric mean to the arithmetic mean of the spectral density. WE ranges from 0 for sinusoidal waveforms to 1 for white noise. For the components shown in Fig. 3A, WE varies between 0.19 and 0.93 (see values given in Fig. 3B). For WE higher than 0.9, the spectrum is visually indistinguishable from a flat one.

We chose 0.9 as the threshold value to declare a component to be broadband noise. On average only 12 components per segment were found to have temporal structure, while the remaining 4 had WE greater than 0.9 (the full distribution of WE is shown in S3 Fig.).

Among the kinematic patterns with temporal structure, we classified the ones occurring repeatedly in distinct flight tests of the same fly, and in different flies. A kinematic pattern was classified as frequently occurring if it was observed in at least 3 out of the 10 analyzed flies. Three supersets of these components can be defined based on the type of their characteristic features:

- Kinematic patterns characterized by prominent activation events in their time course.
- Kinematic patterns characterized by dominance of specific signals in their separating vector.
- Kinematic patterns characterized by spectral density peaks at particular frequencies.

These supersets are not mutually exclusive—a minority of the kinematic patterns belongs to more than one category. The kinematic patterns in each of these supersets can be further divided into several classes. Below we first state (for each type of kinematic pattern) its defining feature. Following this, we discuss additional properties of these kinematic patterns, and list functional interpretations of the stroke deformation modes that they encode. The precise algorithmic criteria for assigning a given component to one of the 7 types are given in S2 Text. The corresponding MATLAB code is available through figshare at http://dx.doi.org/10.6084/m9.figshare.1244993.

A. Kinematic patterns characterized by typical events in the activation time course

**Fig 9:**
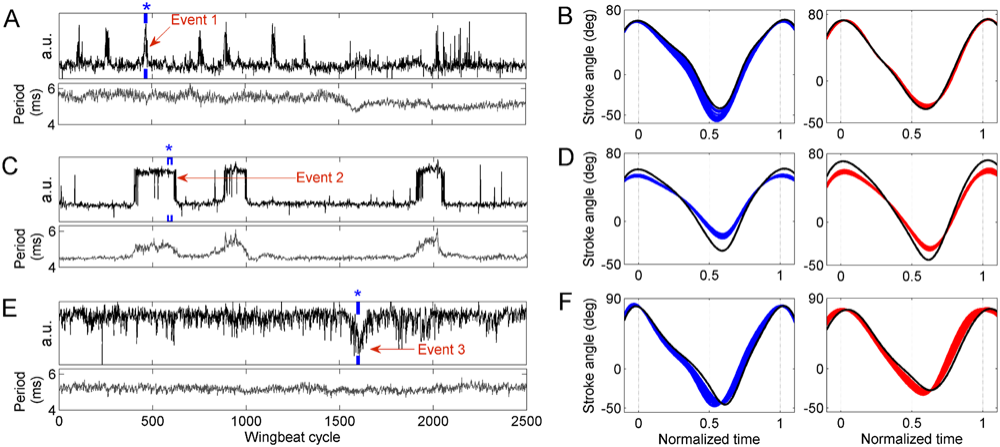
Least-dependent kinematic patterns of types I, II and III. **A)** Activation time course of a type I kinematic pattern (black trace). A typical activation event is marked as Event 1. The lower subpanel shows the wingbeat period (gray trace) during this flight segment. **B)** 10 superposed cycles of wing stroke deformation reconstructed from only the kinematic pattern shown in A (blue: left wing; red: right wing; time window marked with blue bracket and asterisk sign). Black lines show the baseline wing stroke. **C)** Activation time course of a type II kinematic pattern (black trace). A typical activation event is marked as Event 2. The lower subpanel shows the wingbeat period during this flight segment (gray trace). **D)** 20 superposed cycles of wing stroke deformation reconstructed from only the kinematic pattern shown in C (markers as in B). **E)** Activation time course of a type III kinematic pattern (black trace). A typical event is marked as Event 3. The lower subpanel shows the wingbeat period during this flight segment (gray trace). **F)** 10 superposed cycles of wing stroke deformation reconstructed from only the kinematic pattern shown in E (markers as in B).

**Type I:** For these kinematic patterns, the time course is dominated by characteristic events of activation with a time scale of 40 to 100 wingbeat cycles. A typical example is shown in Fig. 9A. Component 1 in Fig. 6 and component 11 in Fig. 3 also belong to this type (the 3 examples were obtained from 3 individual flies). This type of component has a very low correlation with the wingbeat period (Pearson coeff. <0.2).
  The corresponding stroke deformation modes consist of an increase in ventral amplitude for one wing and a simultaneous decrease for the other wing. 10 successive reconstructed stroke cycles during a typical activation event are shown in Fig. 9B. Such a stroke deformation mode is expected to generate yaw torque (see Discussion). The duration (300–500ms) and the form of these events (Fig. 9A) match the time course of yaw torque measured for spontaneous saccades during tethered flight [32,33].

**Type II:** The time course in this type of kinematic patterns is correlated (Pearson coeff. > 0.45) with changes in wingbeat period. Fig. 9C shows such a component, with three sharply defined activation events. During these events only the overall stroke duration changes, while the ratio of downstroke to upstroke duration remains unaltered.
  The corresponding stroke deformation modes involve a symmetric change in stroke amplitude of both wings. 20 successive reconstructed stroke cycles during one activation event are shown in Fig. 9D. Such a stroke deformation is expected to alter the total flight force (lift and/or thrust)—see Discussion.
**Type III:** These kinematic patterns are dominated by activation events with a time scale of 40 to a few hundred wingbeat cycles, during which the ratio of downstroke to upstroke duration is significantly altered. A typical activation time course is shown in Fig. 9E.
  In these kinematic patterns, the stroke deformation may also involve a change in stroke amplitudes. The change in downstroke-to-upstroke ratio and in the amplitude can be bilaterally symmetric or asymmetric. In some cases, this stroke deformation is coupled with a change in wingbeat frequency (S2 Fig.). 10 successive reconstructed stroke cycles during an activation event are shown in Fig. 9F. Such a stroke deformation, if symmetric in both wings, is expected to result in altered pitch torque (see Discussion).

B. Kinematic patterns characterized by the dominance of specific contributing signals

**Fig 10:**
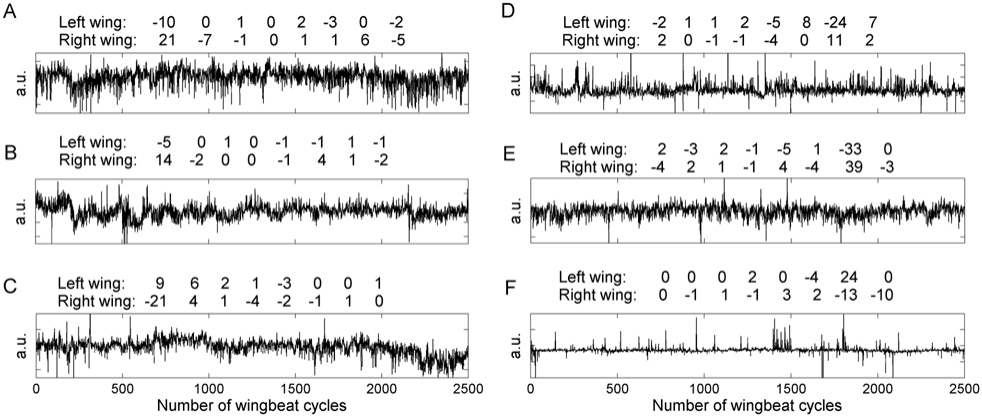
Least-dependent kinematic patterns characterized by specific separating vectors. **A–C)** Activation time course in type IV kinematic patterns. **D–F)** Activation time course in type V kinematic patterns. Examples from 3 different flies are shown for each type. The corresponding separating vectors are printed in two rows for ease of visualization. The vectors are scaled to operate on signals with unit variance (see the main text).

Most of the recurring components are linear combinations that include the majority of the 16 phase points. However, two unusually simple linear combinations were seen to occur repeatedly:

**Type IV:** These LDCs are dominated by the difference of wing stroke positions at dorsal stroke reversal. Time courses of type IV kinematic patterns from 3 flies are shown in Fig. 10A, B and C together with their separating vectors (coefficients of the linear combination).

**Type V:** These LDCs are dominated by the difference of wing stroke positions at mid-upstroke. See Fig. 10D, E, F for examples.
  The separating vectors shown in Fig. 10 are scaled to operate on signals of unit variance so that they directly show the relative contribution of the variability of each phase point in constructing the given LDC. The temporal features appearing in components of type IV and V do not have a consistent pattern. The activation time courses in some of these kinematic patterns are nearly spectrally flat (WE > 0.7). These properties suggest that the kinematic patterns corresponding to these two components might not be involved in flight control (see also Discussion).

C. Kinematic patterns characterized by spectral density peaks at particular frequencies

To characterize the time scales that dominate the activation time course of a given kinematic pattern, it is useful to examine its power spectrum. For most of the frequently recurring kinematic patterns, the Welch spectral density of the time course has high power at low frequencies (<0.05/cycle) and is relatively flat at high frequencies (>0.1/cycle), as in the top three panels in Fig. 3B. Two specific types of deviations from this usual pattern were seen to occur repeatedly and the corresponding kinematic patterns are classified as type VI and VII.

**Fig 11:**
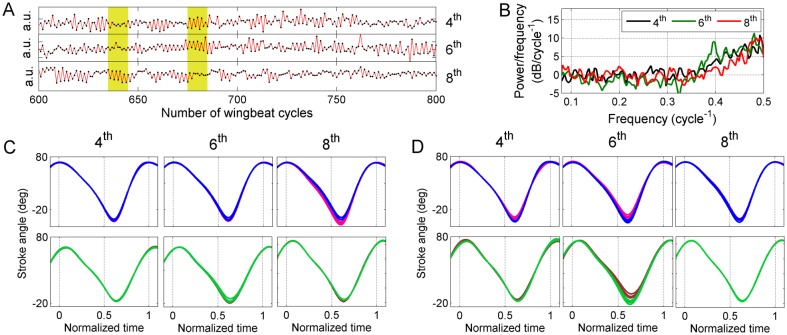
Least-dependent kinematic patterns with 2 cycle periodicity in the activation time course (Type VI). **A)** Activation time course in 3 kinematic patterns of type VI (only 200 wingbeat cycles shown for clarity). **B)** Welch power spectra of the time courses shown in panel A. **C)** 10 consecutive cycles of wing stroke deformation reconstructed from each of the 3 kinematic patterns in A, during the left highlighted interval (upper row: left wing; lower row: right wing). **D)** 10 consecutive cycles of wing stroke deformation during the right highlighted interval in A. Odd-numbered and even-numbered cycles are plotted in different colors. The regular alternation of ventral amplitude between higher and lower values in successive cycles is best visible in C, right column and D, middle column.

**Type VI:** For these kinematic patterns, the power spectrum of the activation time course is dominated by the highest frequencies. The power density increases by about an order of magnitude between frequency 0.35/cycle and 0.5/cycle (the Nyquist frequency). The activation time course contains intermittent intervals during which a period-2 pattern develops: the magnitude increases and decreases in successive cycles. In contrast to other types of kinematic patterns, multiple type VI components frequently co-occurred in a flight segment. As an example, Fig. 11A shows 3 components obtained from a single flight segment. The period-2 patterns do not occur in all three components simultaneously. Fig. 11B shows the power spectra.

The stroke deformation mode of this type encodes significant symmetric changes in ventral amplitude and varying amounts of changes in the rest of the wing stroke. The exact waveform of the wing stroke varies between components. The stroke deformation modes corresponding to the 3 components in Fig. 11A (in the highlighted intervals) are shown in Fig. 11C, D. Their superposition results in somewhat different wing strokes during the two intervals.

**Fig 12:**
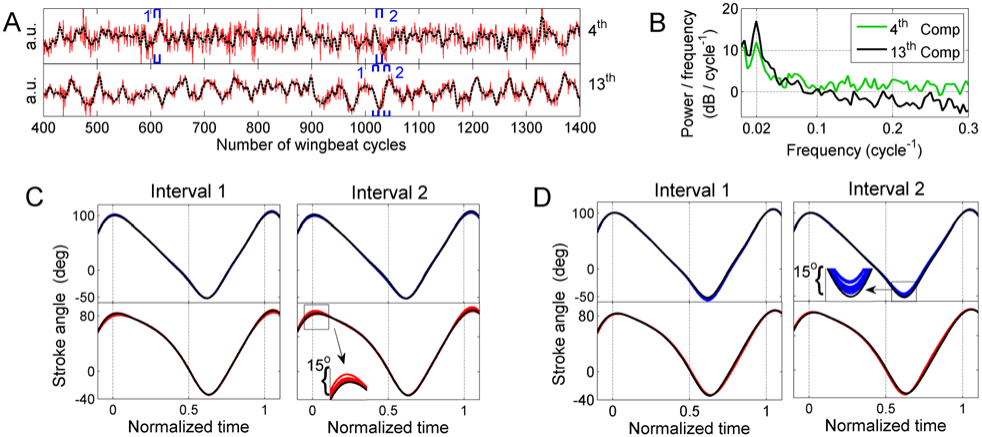
Least-dependent kinematic patterns with 40 to 50 cycle periodicity in the activation time course (Type VII). **A)** Activation time course in two kinematic patterns of type VII, obtained from the same flight segment (black dashed line: low-pass-filtered time course as visual guide). **B)** Welch power spectra of the time courses presented in A. **C)** 10 superposed cycles of wing stroke deformations reconstructed from kinematic pattern 4 (blue: left wing; red: right wing). Interval 1 and interval 2 refer to the time windows marked in the upper subpanel of A. The dorsal amplitude of the reconstructed right wing stroke decreases from the baseline value by about 5° in the 1st interval and increases by a similar amount in the 2nd. The reconstructed left wing stroke does not show any deviation from the baseline wing stroke. **D)** 10 superposed cycles of wing stroke deformations reconstructed from kinematic pattern 13 (otherwise as in C). In this kinematic pattern, the ventral amplitude of the reconstructed left wing stroke increases (in the 1^st^ interval) and decreases (in the 2^nd^ interval) from the baseline value by about 5°.

**Type VII:** For these kinematic patters, the power spectrum of the activation time course has a dominant peak at the frequency of 0.02/cycle. The time course is periodically modulated, with a period of 40–50 wingbeat cycles—see Fig. 12A, B. In the flight segments that contained these components, typically only one or at most two type VII components occurred.

For these kinematic patterns, the stroke deformation modes did not show any commonality. For example, component 4 in Fig. 12A encodes changes in dorsal amplitude whereas component 13 encodes changes in ventral amplitude of the opposite wing (Fig. 12C and D, respectively). Note also that the periodic modulations in these two components are not fully synchronized.

**Fig 13:**
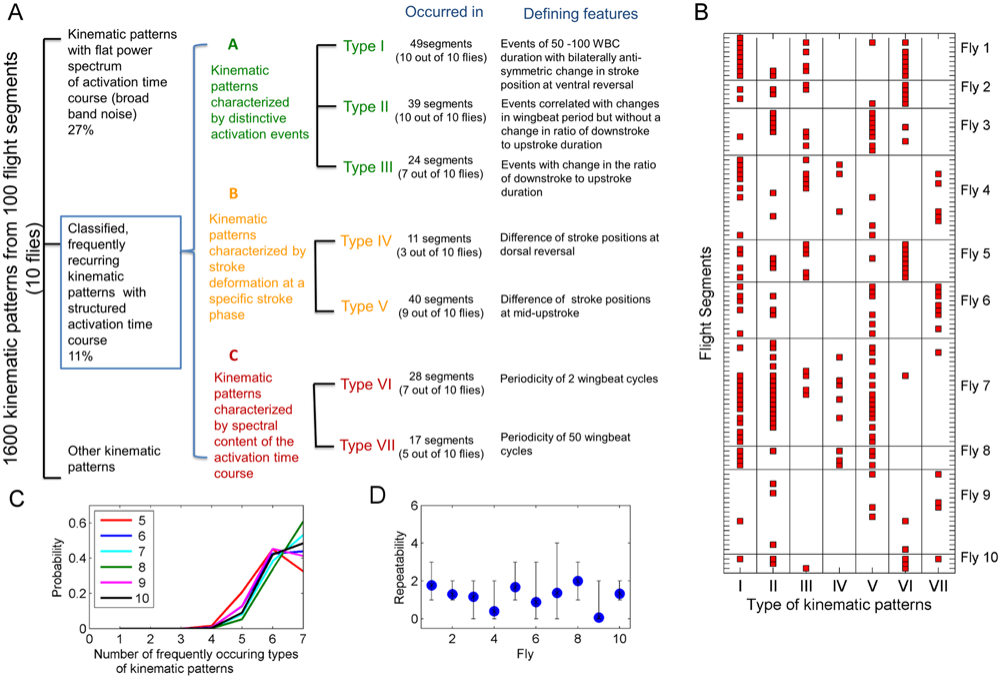
Classification summary and repeatability of kinematic patterns. **A)** 1600 kinematic patterns from 100 flight segments (measured in 10 flies) were first divided into kinematic patterns with and without temporal features in the activation time course. Among kinematic patterns with temporal features, the ones occurring frequently were further classified into 7 types, based on classification criteria fully specified in S2 Text. **B)** Distribution of the 7 classified types of kinematic patterns (filled boxes) among the 100 flight segments. Horizontal lines separate individual flies. **C)** Given the distribution of kinematic patterns in B, the probability of finding a given number of frequently occurring kinematic patterns when a randomly selected sub-sample of the flies is analyzed. Legend indicates the number of flies in the sub-sample. **D)** The number of types of classified kinematic patterns that co-occur in a pair of flight segments from a given fly. Filled circle: the number of co-occurring types averaged over all pairs of flight segments; bar: the range of the number of co-occurring types. For details see text.

Fig. 13A shows a summary of the classification, and gives the number of flies and flight segments in which the kinematic patterns of a particular type were found. Kinematic patterns of types I, II and V are the most commonly occurring ones. Kinematic patterns of type VI, with the striking period-2 pattern of activation, were found in 6 of the 10 examined flies. Fig. 13B gives the full distribution of occurrences of the classified patterns in the 100 analyzed flight segments.

To evaluate the sensitivity of our results to the number of flies analyzed, we examined how many of the patterns in Fig. 13B would be classified as frequently occurring within batches of fewer flies. We obtained batches of 5, 6, 7, 8, 9 or 10 flies by subsampling from the measured set of 10 flies (with repetition allowed). A kinematic pattern was declared frequent if it appeared in at least 25% of the flies in a given batch (e.g., in 2 out of 8 flies or in 3 out of 10 flies). In Fig. 13C, we show the distribution of the number of such frequent patterns in all batches of a given size. It is seen that even in batches of 7 flies, the most frequently found number of classified types of patterns is 7 (identical to the number we obtained from the full set of flies). For batches of 5 flies, however, the most frequently found number of patterns is 6—i.e., had we worked with only 5 flies, we would have likely missed one of our 7 classified types of patterns. (For batches of 6 flies, finding 6 patterns or 7 patterns is approximately equally likely). We cannot exclude that additional types of classified patterns would have been found had we worked with more than 10 flies.

The repeatability of the classified patterns in recordings from an individual fly can be judged from Fig. 13B. To summarize this repeatability, we defined a simple measure as follows. For each pair of flight segments from a given fly, we counted the number of classified types of patterns that occurred in both flight segments. The average of these counts over all pairs of segments from a given fly gives a number between 0 and 7, with 0 indicating no repeatability and 7 perfect repeatability of the classified patterns. These averages, as well as the minimal and maximal counts, are shown individually for all 10 flies in Fig. 13D. It is seen that typically, only 1 or 2 classified types of kinematic patterns are shared by a randomly chosen pair of flight segments. At most 4 types of patterns were found to co-occur in a pair of segments.

## Discussion

### Least-dependent component analysis as a tool for identifying independently controlled kinematic patterns

The goal of our study was to identify kinematic patterns of the wing motion that are controlled independently of each other. Physiologically, these independent kinematic patterns can arise, for instance, from the activity of parallel anatomical pathways. The complete set of such elementary kinematic patterns can be viewed as a basis from which the fly composes its various maneuvers. Our method was designed to identify elementary patterns that are repeatedly activated in the available kinematic dataset, but cannot judge the completeness of the obtained set of patterns.

Independent control of some aerodynamic and kinematic parameters has already been proposed in previous insect flight studies [29,34,35]; for a review refer to [3]. Typically, the independence was assessed by estimating the correlation of these parameters during a flight recording. For example, in the study of Balint and Dickinson for blowflies [29], the downstroke deviation was shown to have no significant correlation with either the dorsal amplitude of the same wing or the wingbeat period.

Uncorrelatedness is a necessary, but not sufficient condition for statistical independence; non-linear statistical dependencies can persist even if the correlation coefficient is zero. Examining a scatter plot of two variables (as in [29]) can help in excluding such non-linear dependencies. For an automatized computational approach, however, a quantitative evaluation is necessary. In our study, we assessed statistical independence using mutual information. This measure captures both linear and non-linear statistical dependencies: two variables are statistically independent if and only if their mutual information is zero.

In contrast to previous studies, we attempted to systematically identify all independently controlled kinematic patterns that occurred in the measured flies. Rather than examining a pre-determined set of kinematic features, we started off from the full wing stroke trajectory and used least-dependent component analysis (LCA) to compute a set of kinematic patterns that have minimal mutual information. Each kinematic pattern was associated with a specific deformation mode of the wing stroke. While for some patterns this deformation can be expressed in terms of a single standard kinematic parameter (such as stroke amplitude), for others the stroke deformation was more complex, involving e.g. example a change in both stroke amplitude and downstroke-to-upstroke ratio. In blowflies, Balint and Dickinson [29] identified the downstroke deviation and the dorsal amplitude as two mutually independently controlled kinematic features, and pointed out that a change in either of these features was closely coupled with changes in other aspects of the wing stroke. Our method *directly* searches for independently activated deformations of the entire wing stroke. Another significant difference compared to Ref. [29] is that our analysis takes into account the motion of *both* wings, and the degree of bilateral (anti)-symmetry is a defining feature of the kinematic patterns we identify.

**Fig 14:**
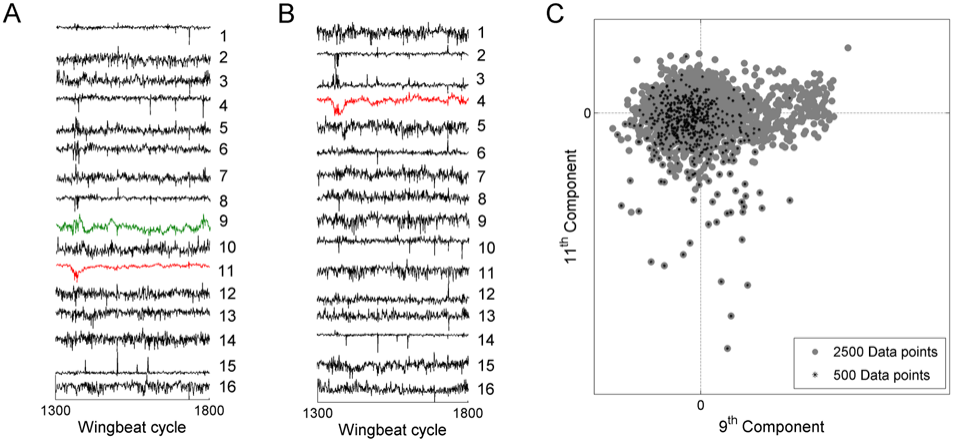
Data length and success of LCA analysis. **A)** Least-dependent components obtained by analyzing a flight segment of 2500 wingbeat cycles (input signals as in Fig. 6A). A segment of 500 wingbeat cycles is shown (cycles 1300 to 1800 in Fig. 6B). Component 9 and component 11 correspond to kinematic patterns of Type II and I, respectively. **B)** Least-dependent components obtained by analyzing the input signals from only this restricted segment of 500 WBCs. LCA failed to separate the type II kinematic pattern from other kinematic variations (there is no analogue of component 9 in A). Component 4 isolates the type I kinematic pattern, but not as cleanly as the analogous component 11 in A. **C)** Scatter plot of component 9 vs. component 11 in the full flight segment (2500 WBCs). The points belonging to the restricted segment (cycles 1300 to 1800) are highlighted in black. It is seen that the short segment does not sufficiently capture the full distribution of the data (the full distribution is more elongated in the x-direction).

Our computational method is based on least-dependent component analysis with explicit evaluation of mutual information. To successfully apply this advanced statistical tool, a sufficiently large sample size is necessary, typically thousands of wingbeat cycles. Long-duration flight recording increases the probability of the fly exerting multiple types of kinematic patterns during the recording, as well as the probability of repeated occurrence of activation events in each kinematic pattern. The former is crucial for identification of patterns that occur mutually independently and the latter enhances the reliability of their separation. In Fig. 14, we show the result of applying LCA to a flight segment of insufficient length (500 cycles). This leads to a failure in separating some of the kinematic patterns that were successfully separated when the analysis was applied instead to a 2500 cycle segment. It is likewise important for the analysis to start from a sufficient number of input signals to LCA. Each signal corresponds to a specific phase point in the wing stroke cycle; a higher number of phase points capture the stroke deformations more precisely. We used 16 phase points (8 for each wing), as we found that working with more points did not yield additional deformation modes with structured time course of activation (but considerably increased the computation time). We illustrate this with an example in S4 Fig., in which one flight segment was analyzed starting from 8, 16, or 24 phase points. Analysis based on a higher number of phase points preserved the classified kinematic patterns obtained with a lower number of points (S4A, C Fig.). The new components obtained from a higher number of phase points do not have well-delineated activation events (in comparison to the classified kinematic patterns), and have a relatively flat power spectrum (Wiener entropy above 0.8)—see S4B, D Fig. The classified stroke deformation modes involve correlated changes in multiple phases of the stroke cycle and hence their occurrence can be inferred by analyzing as few as 4 phase points. Such coherent nature of the wing stroke has been found also in previous fly studies [29]. When applying our method to other types of kinematic data, however, a higher number of sampled phase points may be necessary to uncover the independent kinematic patterns.

Our procedure has some commonalities with principal component analysis (PCA). Both in PCA and LCA, the components are constructed as linear combinations of the input signals. The principal components are uncorrelated, but not necessarily fully statistically independent. PCA has often been used for dimensional reduction, for example in the analysis of human movements (e.g. [27,28]). In animal flight literature, PCA was used to quantify the complexity of bat wing kinematics in [36]. In our study, we did not restrict ourselves to components carrying the dominant contributions to the signal variance; some of the functionally most important deformation modes of the wing stroke occur highly intermittently, and the corresponding component therefore gives only a small contribution to the total variance. Rather, we aimed to identify variations that are fully statistically independent from each other. The framework of LCA, rather than PCA, was therefore appropriate in our case. In S5 Fig., we show an example in which LCA gives significantly different results from PCA. In this case, bilaterally anti-symmetric stroke deformations isolated in one LCA component are distributed in three PCA components, which have high mutual statistical dependence. We systematically compared the mutual dependence of components obtained from LCA to the mutual dependence of components from PCA, in each of the 100 recorded flight segments. Many of the principal components were found to be strongly mutually dependent (S5C Fig.).

Our computational analysis assumed that the elementary kinematic patterns (i.e., the stroke deformations generated by independent neuromotor controls) superpose linearly. This assumption is expected to be satisfied only approximately. On one hand, as the direct steering muscles all attach to the wing hinge, a linear summation of their effects on the wing stroke may be expected. On the other hand, the muscles insert at different sclerites that can to some extent move with respect to each other; this likely leads to nonlinear summation in blowflies ([20] and references therein). A linear superposition of the effects of indirect muscles and direct steering muscles was inferred for *Drosophila* in [37]. The elementary kinematic patterns identified in our analysis adequately represented the wing kinematics both when active one-by-one and when active simultaneously (Fig. 8 and S2 Fig.). This provides a consistency check for the assumption of linear superposition (which was used in identifying the elementary kinematic patterns). A direct test of this assumption would be possible in a stimulated-flight setup, using stimuli that activate the individual kinematic patterns.

### Classification of the least-dependent kinematic patterns and implications for flight control

In most of the flight segments analyzed, LCA decomposed the wing motion into a set of linear components with insignificant or only marginally significant mutual dependence. Each such component is associated with a specific deformation mode of the wing stroke (the terminology is summarized in Table 1). The deformation mode together with the time course of its activation specifies a kinematic pattern of the wing motion. The kinematic patterns defined by components that are statistically independent could, in principle, result from independent neuromotor controls.

Alternatively, some of the obtained statistically independent kinematic patterns may reflect other sources, such as noise in neuromuscular activity, experimental artifacts (e.g., the mistracking in Fig. 5), or variability unrelated to flight control. To narrow down the set of candidates for elementary kinematic patterns (i.e., patterns that are generated by independent neuromotor flight controls), we restricted further analysis to components that (i) had temporal structure significantly distinct from white noise, and (ii) occurred repeatedly in multiple flight segments and in different flies. The first criterion was motivated by our expectation that the activation course of the elementary patterns will contain time scales similar to those seen in various flight maneuvers (i.e., between several wing stroke cycles and hundreds of cycles). Components with a flat power spectrum, on the other hand, are more likely to result from physiological noise or from measurement artifacts. The second criterion required the pattern coded by the component to occur in at least 3 of the 10 analyzed flies. We cannot exclude that some of the infrequently obtained independent components do represent elementary patterns. The rarely obtained components can, however, also arise e.g. from transient nonlinear couplings between elementary patterns, or from statistical limitations (limited duration of the recorded flight segment). In our search for the elementary kinematic patterns, we therefore chose not to classify the rarely occurring components. The described elimination may be viewed as a dimensional reduction; its goal, however, was to construct a lower-dimensional space that still contains the frequently activated independent kinematic patterns, rather than best approximating the time course of the original signal.

The resulting classification of kinematic patterns is summarized in Fig. 13. We identified 7 types of frequently recurring patterns. For five of these types, the kinematic pattern was found in at least 6 of the 10 examined flies. As our analysis was based on recordings of unstimulated flight of limited duration, we do not view as surprising that some kinematic patterns were not observed in all flies. To help in deciding which of the 7 frequently recurring types of patterns should be viewed as elementary kinematic patterns, we evaluated their possible functional roles.

To functionally interpret a given kinematic pattern, we first examined the stroke deformation mode associated with it. Previous studies [8,9,13] used dynamically scaled robotic models to establish the correspondence between changes in wing stroke kinematics and changes in aerodynamic forces. The full 3-dimensional kinematics of each wing is specified by the time course of three angles: the stroke position, the morphological angle of attack, and the deviation. In our study, only the stroke position was measured. We therefore could not directly evaluate the aerodynamic forces and their moments, as was done in [8,29]. However, previous studies with optically stimulated tethered fruit flies [38] established a linear relation between the difference of stroke amplitudes in the two wings and the yaw torque generated by the fly; likewise, the difference of stroke amplitudes is correlated with yaw torque during a free flight saccade [13]. We therefore concluded that the wing stroke deformation typical for type I kinematic patterns (i.e., a bilaterally antisymmetric change in stroke amplitude and no change in wing period) is associated primarily with a change in yaw torque. For the type II kinematic patterns, the stroke deformation mode involves a bilaterally symmetric change in stroke amplitude coupled with a change in wingbeat period. A similar stroke deformation can be evoked by optical stimulation (vertical movement of the background pattern) and results in a change of total flight power [12]. For the type III kinematic pattern, the stroke deformation involves a change in the ratio of downstroke duration to upstroke duration. When symmetric in both wings, this change in stroke trajectory is expected to alter primarily the pitch torque acting on the body. In Ref. [9], the aerodynamic output was evaluated for wing strokes recorded in hovering free flight and in tethered flight; these wing strokes differed primarily by the downstroke-to-upstroke ratio (1.16 in free flight vs.1.53 in tethered flight). It was shown that in the tethered case, the wing stroke generates a strong pitch torque (that would cause an untethered fly to pitch nose-down by 20° after a single stroke cycle) [9]. In our flight tests, the downstroke-to-upstroke ratio was in the range 1.45–1.55 for the baseline wing stroke, but decreased to values as low as 1.1 when a type III kinematic pattern was activated. We therefore associate the type III kinematic patterns with a strong change in pitch torque.

The inferences given above were based on the similarity of stroke position trajectory in the stroke deformations identified by us and in the kinematic changes analyzed in previous literature. We cannot exclude that there are differences in the morphological angle of attack or in stroke deviation that would modify the torques or forces acting on the body. In Ref. [29] kinematic changes in the three rotational degrees of freedom were found to be mutually strongly coupled, resulting in concerted modifications of the entire wing stroke. It is, however, possible that the deformation of the stroke position trajectory (even when sampled at a high rate) is not fully indicative of the full 3-dimensional wing kinematics.

To further judge the functional relevance of the kinematic pattern of a specific type, we next compared the typical features in its activation time course to the typical time courses of known flight maneuvers. For the type I kinematic pattern, the time course of the activation events matches the time course reported for fictive saccades induced by visual expansion stimulus in tethered flight [32,33]. The type I kinematic pattern can therefore be identified with activations of the saccade motor program (see [39] for a discussion of the relation between tethered and free flight saccades).The activation events for the kinematic patterns of type II and III are typically of longer duration, consistent with the time scales on which *Drosophila* is known to control flight power and pitch.

In type VI kinematic patterns, the ventral amplitude for both wings in turn increases and decreases in successive wing strokes; such regular switching persists for dozens of cycles. It is plausible that this kinematic pattern is caused by some steering muscle(s) becoming active in every other wingbeat cycle. Such a pattern of activity of the b1 steering muscle, lasting for 16 cycles, was recorded in the blowfly by Balint and Dickinson [20] (the first 0.1 sec in their Fig. 6A), and was correlated with a period-2 pattern in the downstroke deviation (known to be highly correlated with ventral amplitude). In *Drosophila*, activation of the M.b2 and M.I1 steering muscles with average frequency of 2 to 3 wingbeat cycles was recorded in [19] (their Fig. 6B). As we recorded only wing kinematics and not muscle activity, we cannot identify which steering muscle(s) are responsible for the type VI kinematic pattern. It appears unlikely that this pattern has a direct functional role in flight control, yet the pattern occurred frequently and was observed in the majority of the examined flies.

Kinematic patterns of types IV and V involve a bilaterally antisymmetric change in the dorsal reversal positions (type IV) or in the stroke angles at the mid-upstroke phase of the stroke cycle (type V). The activation time courses in these kinematic patterns do not have prominent temporal features, and we have not been able to relate these patterns to any known flight maneuver. It is possible that these two types of kinematic pattern simply reflect particular sources of noise in neuromuscular activity. In this case, the fly would control the bilaterally symmetric variations in dorsal amplitudes (which are prominent in type II kinematic patterns), but not the antisymmetric variations (which are usually of lower magnitude). In blowflies [29], dorsal amplitude was found to vary independently of downstroke deviation. Because the (3d) kinematics of an individual wing was studied in Ref. [29], a direct comparison to our findings on stroke deformations evaluated jointly for both wings is difficult.

The type VII kinematic pattern, which was obtained as a separate component in 8 flight segments, is characterized by a slow periodic modulation of the wing stroke (with a period of 40–50 wingbeat cycles). In other flight segments, however, such periodic modulation was not isolated by LCA into a separate component, but rather remained mixed with other kinematic patterns (predominantly of type I). This indicates that the type VII kinematic pattern may be nonlinearly coupled with other pattern types. We consequently do not include it among the elementary kinematic patterns that form a linear basis for the construction of the total wing stroke. The type VII kinematic pattern may be related to the yaw torque fluctuations with similar periodicity that were reported by Heisenberg and Wolf [40].

Based on the properties described above, we propose that the kinematic patterns of types I, II, III and VI are elementary kinematic patterns. We view these 4 elementary patterns as part of the basis, from which the total deviation of the wing kinematics from the baseline stroke is composed by linear superposition. For each of these 4 kinematic patterns, the corresponding stroke deformation modes are typically activated intermittently, with well-delineated activation events separated by intervals of relative inactivity. The activation events of the 4 elementary patterns occurred both one-at-a-time (demonstrating that the 4 corresponding neuromotor controls are not coupled by strong mutual excitation) as well as simultaneously (demonstrating that they are not coupled by strong mutual inhibition).

We cannot exclude that there are additional elementary kinematic patterns, which were not activated frequently during our measurements, or which are not activated at all during tethered, unstimulated flight. It is also possible that some of the elementary kinematic patterns that we identified result from the simultaneous activation of several neuromotor controls that may act mutually independently in other behavioral settings. Our finding of 4 elementary kinematic patterns therefore gives only a lower bound for the number of independent neuromotor controls.

Our results have a partial correspondence to the control system implemented in the simulations of Dickson et al. [41]. In their integrative model of *Drosophila* flight, the navigation through a virtual environment was achieved by the appropriate activation of four “deformation modes”: the pitch mode, the yaw mode, the roll mode, and the throttle mode. Each mode consists of a suitable deformation of the wing stroke that achieves the required change in flight torque or force. The activation of each of these modes is achieved by a separate controller. The yaw mode and the throttle mode defined in Ref. [41] are in direct correspondence to the kinematic patterns of type I and II we found in our study. The pitch mode in Ref. [41] is functionally similar to the kinematic pattern of type III, but consists of a different deformation of the wing stroke. The deformation modes in Ref. [41] were designed *a priori*, based on previous conceptions of *Drosophila* flight control. In contrast, in our study we extracted the independently controlled deformation modes of type I, II and III from an automatized computational analysis of unstimulated flight recordings. Our results thus give support to the control framework of Dickson et al.

## Conclusion

In this study, we developed a new method for the analysis of rhythmic movement, and applied it to high-speed measurements of wing kinematics in tethered flying fruit flies. The method is designed to identify the elementary kinematic patterns, i.e., independently controlled kinematic changes that combine to generate the modulations of the wing stroke during flight maneuvers. It is based on a systematic search for linear components of the recorded signal that are as close as possible to mutual statistical independence, as assessed by the mutual information measure.

The computational method we presented is particularly suited for exploratory data analysis. It does not depend on any prior knowledge of expected types of kinematic changes, and proceeds in an unsupervised manner. Given the wide range of wing kinematics patterns in flies, it can be difficult to identify novel patterns by visual inspection of the recorded data. Our method isolates such kinematic patterns in separate components, which facilitates their identification and interpretation. For instance, the periodic patterns identified by us are frequently superposed on other types of kinematic variations, and may be missed without computationally processing the kinematic data. Our method relies on iterative optimization procedures, but its computational cost is sufficiently low to permit automatized analysis of long flight recordings.

Using this method, we identified 7 types of kinematic patterns that recurred frequently in recordings from 10 flies. Four of these types were judged to be elementary kinematic patterns arising from independent neuromotor controls. The findings imply mutually independent control of wing stroke deformations that generate (i) torque about the yaw axis, (ii) torque about the pitch axis, and (iii) total flight force.

The elementary kinematic patterns reflect mutually independent activations in the fly’s neuromotor apparatus. Each pattern may be due to the activity of a single muscle or to a synergy of multiple muscles. Identification of the corresponding muscles requires electrophysiological studies. Our kinematics-based approach, however, has the advantage of studying the neuromotor system in its entirety, and is readily extendable to kinematic data obtained from free flight recordings.

## Supporting Information

S1 Text Summary of the MILCA algorithm for least-dependent component analysis. (DOCX)

S2 Text Algorithmic criteria for assigning the components to the 7 types of kinematic patterns in Fig. 13. (DOCX)

S1 Fig Simultaneously active stroke deformation modes of type I and type III kinematic patterns. **A)** Activation time course of a type I kinematic pattern, with typical spiky activation events. **B)** Activation time course of the type III kinematic pattern in the same flight segment, with a long-duration activation event lasting from cycle 1100 to cycle 2000. Note that some activation events in A occur simultaneously with the long-duration event in B. **C)** Time course of the wingbeat period, which is not correlated with the activation time courses in A and B. **D)** 10 consecutive reconstructed stroke cycles (from the time window marked with blue bracket and asterisk sign in A) with only the type I stroke deformation mode included (blue: left wing; red: right wing). Black lines show the baseline wing stroke. **E)** 10 consecutive reconstructed stroke cycles (from the time window marked with blue bracket and asterisk sign in B) with only the type III stroke deformation mode included.
(TIF)

S2 Fig Co-occurrence of three elementary kinematic patterns in one flight segment. **A–C)** Activation time courses of type I (panel A), type II (panel B) and type III (panel C) kinematic patterns obtained from LCA analysis of one flight segment. **D)** Time course of the wingbeat period, correlated with B and C. **E-G)** 20 consecutive reconstructed stroke cycles with only the type I (panel E), type II (panel F) and type III (panel G) stroke deformation modes included (blue: left wing; red: right wing). Black lines show the baseline stroke cycle. The time window for which the reconstructed stroke cycles are shown is marked with blue brackets and asterisk in panel A (for strokes in E), panel B (for strokes in F) and panel C (for strokes in G).
(TIF)

S3 Fig Distribution of Wiener entropy for input signals and for their least-dependent components. **A)** Histogram of number of least-dependent components per segment that have Wiener entropy less than 0.9 (obtained from 120 flight segments). The average number of such components per segment was found to be 12. **B)** Histogram of Wiener entropy of all (16x120) analyzed signals (upper panel) and least-dependent components (lower panel). Only 1% of signals have Wiener entropy greater than 0.9 (i.e. have flat power spectra) as compared to 26% of least-dependent components. LCA analysis has thus separated broadband noise from significant temporal features.
(TIF)

S4 Fig Consequences of decreasing or increasing the number of analyzed phase points. **A)** Correlation coefficients of the LDCs that were obtained from the analysis of 16 phase points (8 for each wing) with LDCs obtained from the analysis of 8 phase points (4 for each wing). 4 of the components from analysis based on 16 phase points are classified kinematic patterns (component 16 is of type I, component 1 of type II, and components 13 and 15 of type III). Each of these 4 components has very high correlation (marked with arrows) with one of the components obtained from analysis based on 8 phase points. **B)** The time course of components 4, 9, and 10. These components have no correspondence in the analysis based on 8 phase points (white boxes in A). Wiener entropy (WE) values are shown in the legends. The separating vectors (given in the same format as in Fig. 10) indicate that the corresponding stroke deformations are predominantly localized near a specific phase of the cycle. **C)** As in A, but showing correlation coefficients with components that were obtained from the analysis of 24 phase points (12 for each wing). The 4 classified components are again reproduced (white arrows). **D)** Time courses and separating vectors of 5 components (marked with white boxes in C) that have no correspondence in the components obtained from 16 phase points.
(TIF)

S5 Fig Principal components and their mutual dependence. **A)** Time course of the principal components of signals in Fig. 6A. The sharp events dominating principal component 3 are also present in principal component 4—in contrast to the isolation of these events in only one least-dependent component (Fig. 6B). **B)** Dependency matrix of the principal components shown in A. The color code indicates the value of mutual information for a given pair of components. (The value on the diagonal is undefined.) Components with mutual information above 0.26 nats (threshold of significant mutual dependence) are frequent, and 5 pairs have very high mutual information (>0.9 nats). **C)** Distribution of pairwise mutual information, for principal components (solid blue line) and least-dependent components (solid red line) from each flight segment, over a total of 100 flight segments. The dashed black line marks the α = 0.01 confidence limit for rejecting the null hypothesis of zero mutual information. Only 56% of principal component pairs have mutual information less than 0.026 nats, in contrast to 88% of least-dependent components.
(TIF)
