# Correction: Independently Controlled Wing Stroke Patterns in the Fruit Fly *Drosophila melanogaster*

**DOI:** 10.1371/journal.pone.0124475

**Published:** 2015-04-13

**Authors:** Soma Chakraborty, Jan Bartussek, Steven N. Fry, Martin Zapotocky

The following information is missing from the Data Availability section: The preprocessed kinematic data, the MATLAB code for computational analysis, and the analysis results are available through Figshare at http://dx.doi.org/10.6084/m9.figshare.1244993.

There is an error in the Author Contributions. The correct contributions are: Conceived and designed the experiments: SNF MZ. Performed the experiments: JB SC. Analyzed the data: SC MZ JB. Contributed reagents/materials/analysis tools: SNF JB SC. Wrote the paper: SC MZ JB SNF. Designed the computational analysis: MZ SC.

There is an error in the penultimate sentence of the second paragraph of the “The least-dependent components are statistically nearly independent” subsection of the Results. The correct sentence is: The distribution of this null estimate calculated from 100 flight segments (12000 pairs of LDCs) is shown in Fig. 7B (red dashed line).
