# Supplementary material for: Independently Controlled Wing Stroke Patterns in the Fruit Fly Drosophila melanogaster
Source: PLoS One. 2015 Feb 24;10(2):e0116813. doi: 10.1371/journal.pone.0116813 (PMC4339832; doi:10.1371/journal.pone.0116813)
Supplement: S1 Text — (DOCX) [file pone.0116813.s001.docx]

# Summary of the MILCA algorithm

# for least-dependent component analysis

Similarly to standard ICA algorithms (e.g., FastICA [S1,S2]), MILCA starts by transforming the set of 16 input signals **X**=[*X_1_,…, X_16_*] to a set of mutually uncorrelated signals **Y**=[Y_1_,…,Y_16_]:

$\mathbf{Y}=\mathbf{W}_{0}\mathbf{X}$ such that $\mathrm{Cov}(Y_{i},Y_{j})=\left\{_{0\text{ if i}\neq\text{j}}^{1\text{ if i=j}}\right..$ (A1)

This transformation is achieved by suitable rescaling of the principal components of **X**, and is called whitening. **W_0_** is the whitening matrix. Thus, the first step extracts linearly independent (i.e., uncorrelated) components of **X**.

The second step minimizes non-linear dependencies pairwise within the whitened multivariate signal **Y**. The whitened signals are remixed by rotating each pair (*Y_i_, Y_j_*) in its own plane (_16_C_2_ = 120 such pairs in our case) and finding the angle *ϕ_ij_* at which the mutual information (Eq.2 in the main text) of the rotated signals is minimized. The result is

$\left(Y_{1}\ldots Y'_{i}\ldots Y'_{j}\ldots Y_{16}\right)$=$\left(Y_{1}\ldots Y_{i}\ldots Y_{j}\ldots Y_{16}\right),$ (A2)

where **R***_ij_*(*ϕ_ij_*) is a 16×16 rotation vector acting only in the 2×2 subspace (*Y_i_, Y_j_* ). The product of such pairwise rotations for all 120 pairs generates the 16x16 rotation matrix:.

The transformation $\mathbf{Z}=\mathbf{RY}$ defines a candidate set of components. The joint mutual information (Eq.3 in the main text) of the 16 components *Z_i_* is then evaluated, and pairwise remixing of the components is repeated until the joint mutual information converges to a minimum value.

The overall transformation from the set $\mathbf{X}$of 16 time series of phase points to the set $\mathbf{Z}$of 16 least-dependent components is given by

$\mathbf{Z}=\tilde{\mathbf{R}}\mathbf{Y}=\tilde{\mathbf{R}}\mathbf{W}_{0}\mathbf{X}=\mathbf{WX},$ (A3)

where $\tilde{\mathbf{R}}$is the total rotation matrix after the iterative remixing, and $\mathbf{W}=\tilde{\mathbf{R}}\mathbf{W}_{0}$ is the separating matrix. For further details on the MILCA algorithm refer to [S3].

# References

S1. Hyvarinen A (1999) Fast and robust fixed-point algorithms for independent component analysis. IEEE Trans Neural Netw 10: 626–634. doi:10.1109/72.761722.

S2. Hyvarinen A, Karhunen J, Oja E (2001) Independent Component Analysis. S.l: Wiley-Interscience.

S3. Stögbauer H, Kraskov A, Astakhov SA, Grassberger P (2004) Least-dependent-component analysis based on mutual information. Phys Rev E 70: 066123. doi:10.1103/PhysRevE.70.066123.
