# Supplementary material for: Independently Controlled Wing Stroke Patterns in the Fruit Fly Drosophila melanogaster
Source: PLoS One. 2015 Feb 24;10(2):e0116813. doi: 10.1371/journal.pone.0116813 (PMC4339832; doi:10.1371/journal.pone.0116813)
Supplement: S2 Text — (DOCX) [file pone.0116813.s002.docx]

**Criteria for assigning the least-dependent components**

**to the 7 types in Fig. 13**

First, components with near-flat spectral density were eliminated: The spectral density of a given LDC was estimated using the Welch method (Hamming window of length 256 cycles, 50% overlap). The Wiener entropy (i.e., the ratio of geometric to arithmetic mean) of the spectral estimate was computed. Components with Wiener entropy ≥ 0.9 were dropped from further consideration.

Components with Wiener entropy < 0.9 were tested for the criteria listed below. When multiple criteria are listed for a given type, these must be satisfied simultaneously. Low-pass filtering was implemented with idealfilter from MATLAB 7.11 (R2010b). The “Reconstructed wing stroke” refers to the wing stroke reconstructed from the classified component (see Computational Methods in the main text). “Baseline” refers to the wing stroke averaged over the whole flight segment. The separating vectors in the criteria for type IV and V are rescaled so they operate on signals with unit variance (similarly to Fig.10).

**Type I**

1. At least one saccade-like event occurs during the flight segment: The low-pass filtered (cutoff frequency 0.1 cycle^-1^) bilateral difference of ventral amplitudes in the reconstructed wing stroke has at least one peak with the following properties: a) peak height at least 7 degrees, b) half-width in the range 10 cycles to 50 cycles, c) decay to 1/10^th^ of peak height within 100 cycles on both sides of the peak.
2. The ventral amplitudes are bilaterally anti-symmetric or strongly asymmetric: The Pearson correlation coefficient of the low-pass filtered (cutoff frequency 0.1 cycle^-1^) left and right ventral amplitude in the reconstructed wing stroke is between -0.8 and -1, or the variance of the ventral amplitude in one wing is less than 1/10^th^ of the variance of ventral amplitude in the other wing.

**Type II**

- 1. The LDC is correlated with stroke period: The Pearson correlation coefficient of the LDC with the stroke period has absolute value ≥ 0.45.
  2. The upstroke-to-downstroke ratio does not strongly vary: The following condition holds for the low-pass filtered series (cutoff frequency 0.05 cycle^-1^) of the upstroke-to-downstroke ratio of the reconstructed wing stroke: in at least 99% of the wing strokes, the value does not deviate from the baseline by more than 8.5%.

**Type III**

The upstroke-to-downstroke ratio strongly varies: The following condition holds for the low-pass filtered series (cutoff frequency 0.05 cycle^-1^) of the upstroke-to-downstroke ratio of the reconstructed wing stroke: in at least 2% of the wing strokes, the value deviates from the baseline by more than 10%.

**Type IV**

The LDC is dominated by the bilateral difference of wing stroke positions at dorsal stroke reversal:

1. The 1^st^ weight in the rescaled separating vector is greater than twice the maximum of 3^rd^ to 8^th^ weights (in absolute value).
2. The 9^th^ weight in the rescaled separating vector is greater than twice the maximum of 11^rd^ to 16^th^ weights (in absolute value).
3. The 1^st^ and 9^th^ weights in the rescaled separating vector have opposite signs.

**Type V**

The LDC is dominated by the bilateral difference of wing stroke positions at mid-upstroke:

1. The 7^th^ weight in the rescaled separating vector is greater than twice the maximum of 1^st^ to 6^th^ weights (in absolute value).
2. The 15^th^ weight in the rescaled separating vector is greater than twice the maximum coefficient of 9^th^ to 14^th^ weights (in absolute value).
3. The 7^th^ and 15^th^ weights in the rescaled separating vector have opposite signs.

**Type VI**

The power spectrum of the LDC is peaked at the frequency of 1/(2 cycles): The estimated spectral density of the unfiltered LDC increases by at least 6 dB between frequency 0.33 cycle^-1^ and 0.5 cycle^-1^ (assessed from the best linear fit of log power vs. frequency in this band).

**Type VII**

The power spectrum of the LDC is peaked near the frequency of 1/(50 cycles): The highest peak of the estimated spectral density of the unfiltered LDC is at a frequency of 0.018 cycle^-1^ to 0.024 cycle^-1^.
