# Supplementary figures and images for: Independently Controlled Wing Stroke Patterns in the Fruit Fly Drosophila melanogaster
Source: PLoS One. 2015 Feb 24;10(2):e0116813. doi: 10.1371/journal.pone.0116813 (PMC4339832; doi:10.1371/journal.pone.0116813)

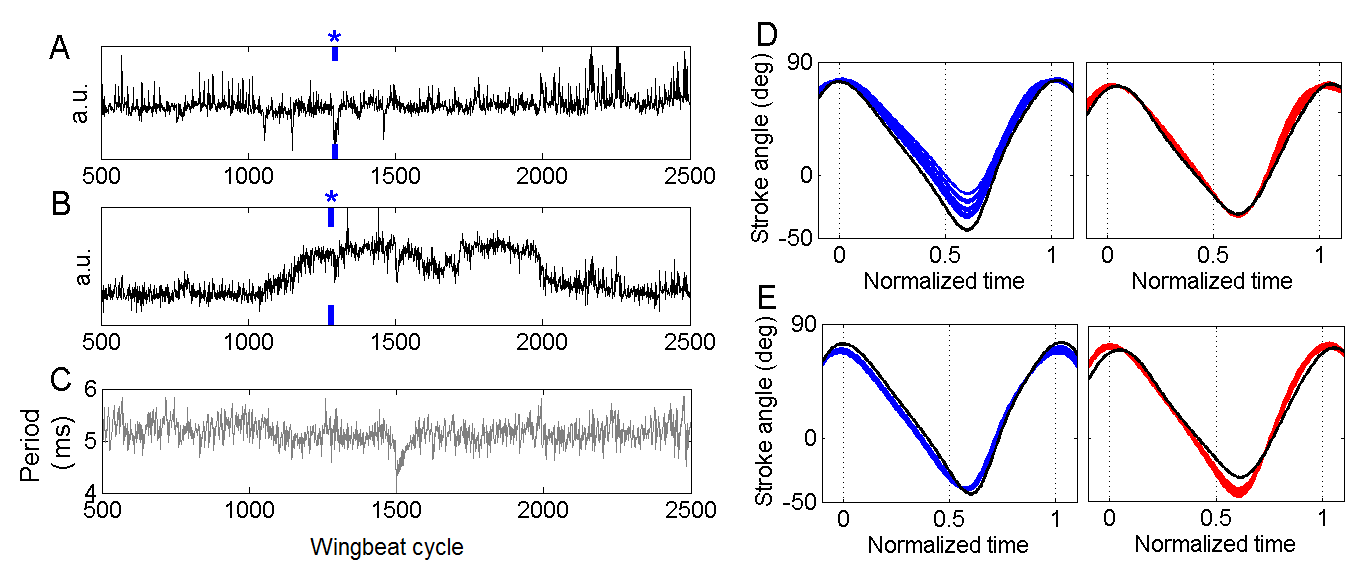

Supplement: S1 Fig — A) Activation time course of a type I kinematic pattern, with typical spiky activation events. B) Activation time course of the type III kinematic pattern in the same flight segment, with a long-duration activation event lasting from cycle 1100 to cycle 2000. Note that some activation events in A occur simultaneously with the long-duration event in B. C) Time course of the wingbeat period, which is not correlated with the activation time courses in A and B. D) 10 consecutive reconstructed stroke cycles (from the time window marked with blue bracket and asterisk sign in A) with only the type I stroke deformation mode included (blue: left wing; red: right wing). Black lines show the baseline wing stroke. E) 10 consecutive reconstructed stroke cycles (from the time window marked with blue bracket and asterisk sign in B) with only the type III stroke deformation mode included. (TIF) [file pone.0116813.s003.tif]

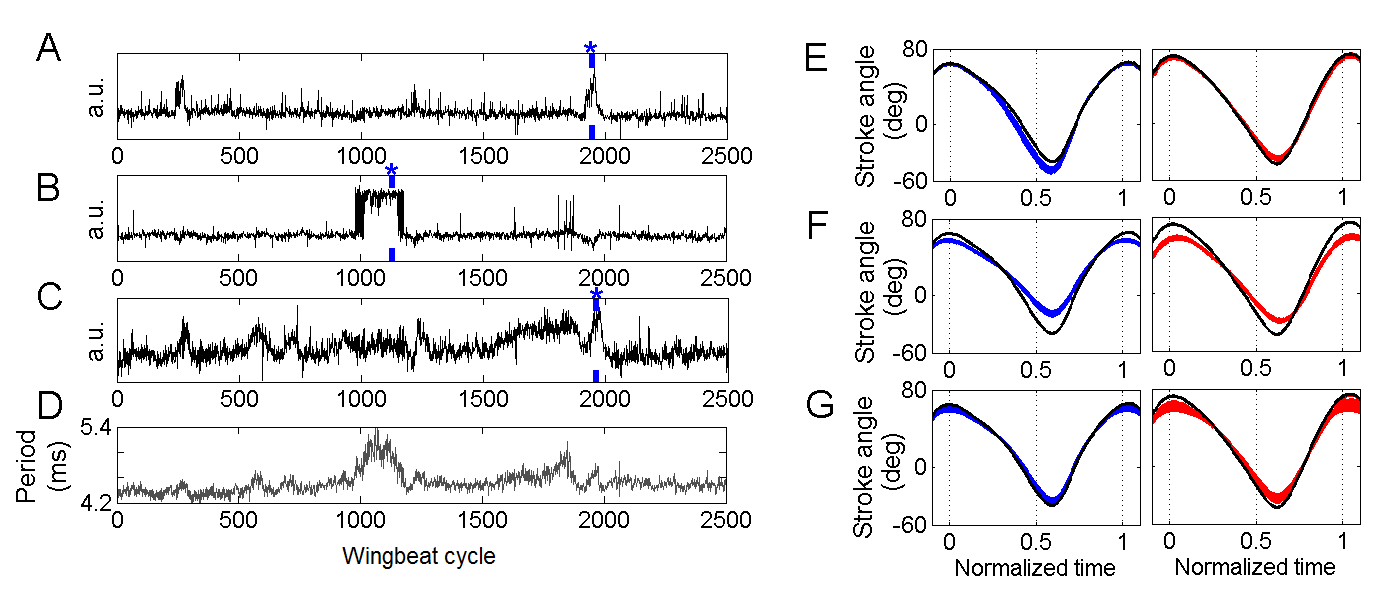

Supplement: S2 Fig — A–C) Activation time courses of type I (panel A), type II (panel B) and type III (panel C) kinematic patterns obtained from LCA analysis of one flight segment. D) Time course of the wingbeat period, correlated with B and C. E-G) 20 consecutive reconstructed stroke cycles with only the type I (panel E), type II (panel F) and type III (panel G) stroke deformation modes included (blue: left wing; red: right wing). Black lines show the baseline stroke cycle. The time window for which the reconstructed stroke cycles are shown is marked with blue brackets and asterisk in panel A (for strokes in E), panel B (for strokes in F) and panel C (for strokes in G). (TIF) [file pone.0116813.s004.tif]

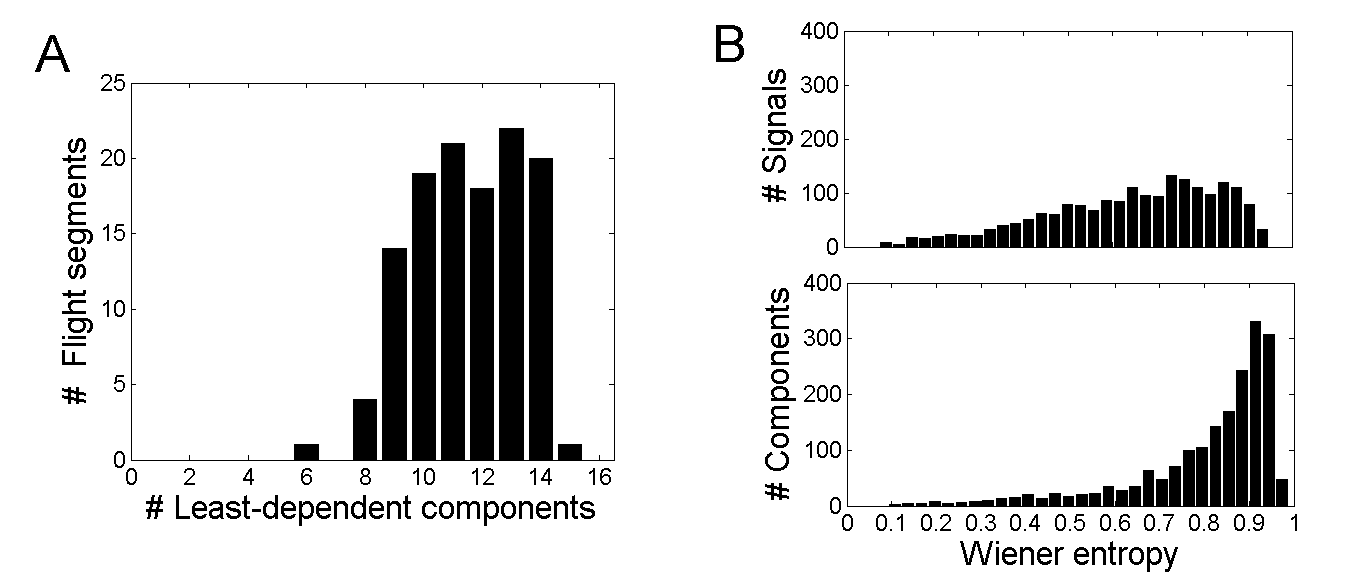

Supplement: S3 Fig — A) Histogram of number of least-dependent components per segment that have Wiener entropy less than 0.9 (obtained from 120 flight segments). The average number of such components per segment was found to be 12. B) Histogram of Wiener entropy of all (16x120) analyzed signals (upper panel) and least-dependent components (lower panel). Only 1% of signals have Wiener entropy greater than 0.9 (i.e. have flat power spectra) as compared to 26% of least-dependent components. LCA analysis has thus separated broadband noise from significant temporal features. (TIF) [file pone.0116813.s005.tif]

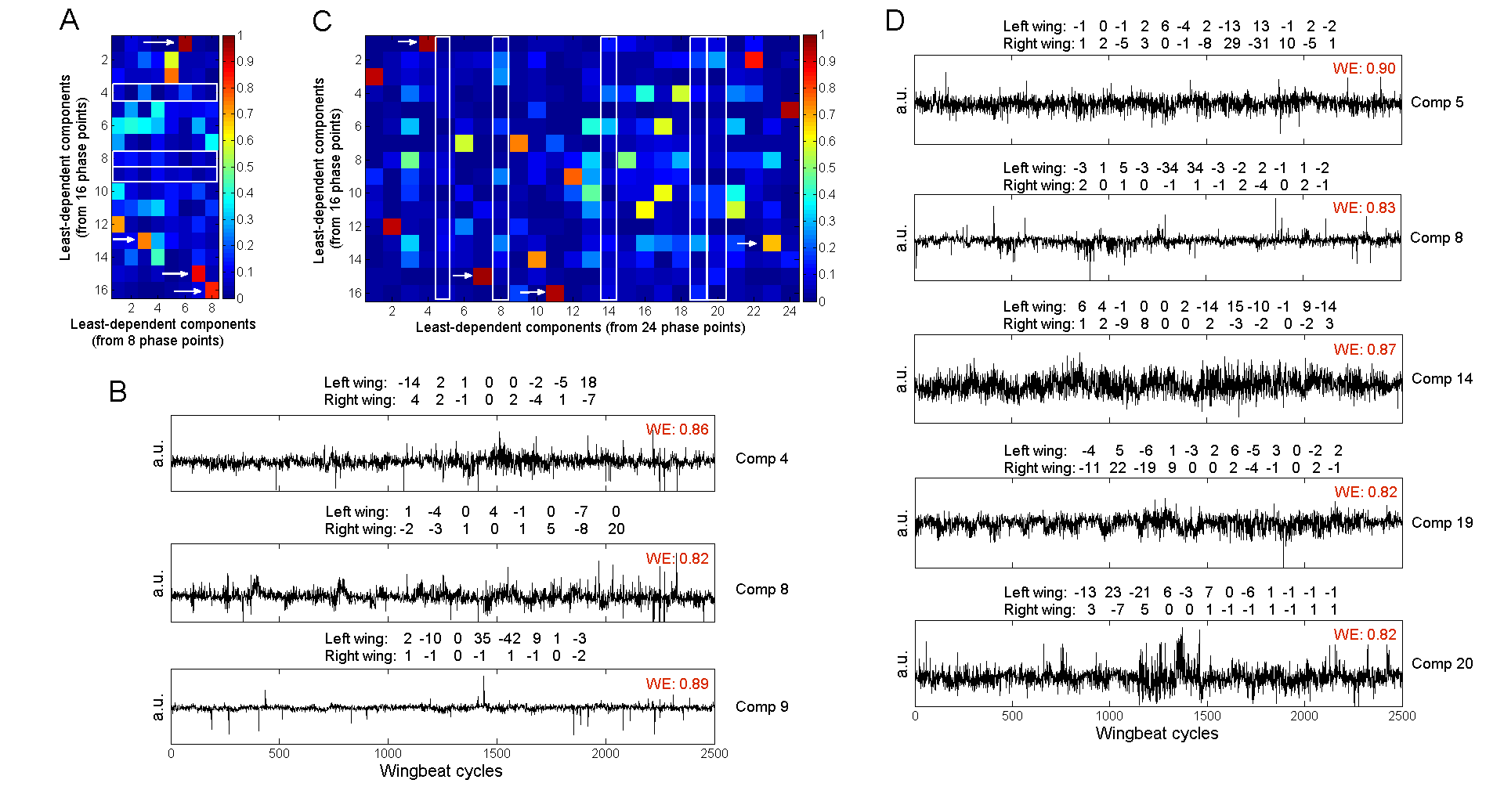

Supplement: S4 Fig — A) Correlation coefficients of the LDCs that were obtained from the analysis of 16 phase points (8 for each wing) with LDCs obtained from the analysis of 8 phase points (4 for each wing). 4 of the components from analysis based on 16 phase points are classified kinematic patterns (component 16 is of type I, component 1 of type II, and components 13 and 15 of type III). Each of these 4 components has very high correlation (marked with arrows) with one of the components obtained from analysis based on 8 phase points. B) The time course of components 4, 9, and 10. These components have no correspondence in the analysis based on 8 phase points (white boxes in A). Wiener entropy (WE) values are shown in the legends. The separating vectors (given in the same format as in Fig. 10) indicate that the corresponding stroke deformations are predominantly localized near a specific phase of the cycle. C) As in A, but showing correlation coefficients with components that were obtained from the analysis of 24 phase points (12 for each wing). The 4 classified components are again reproduced (white arrows). D) Time courses and separating vectors of 5 components (marked with white boxes in C) that have no correspondence in the components obtained from 16 phase points. (TIF) [file pone.0116813.s006.tif]

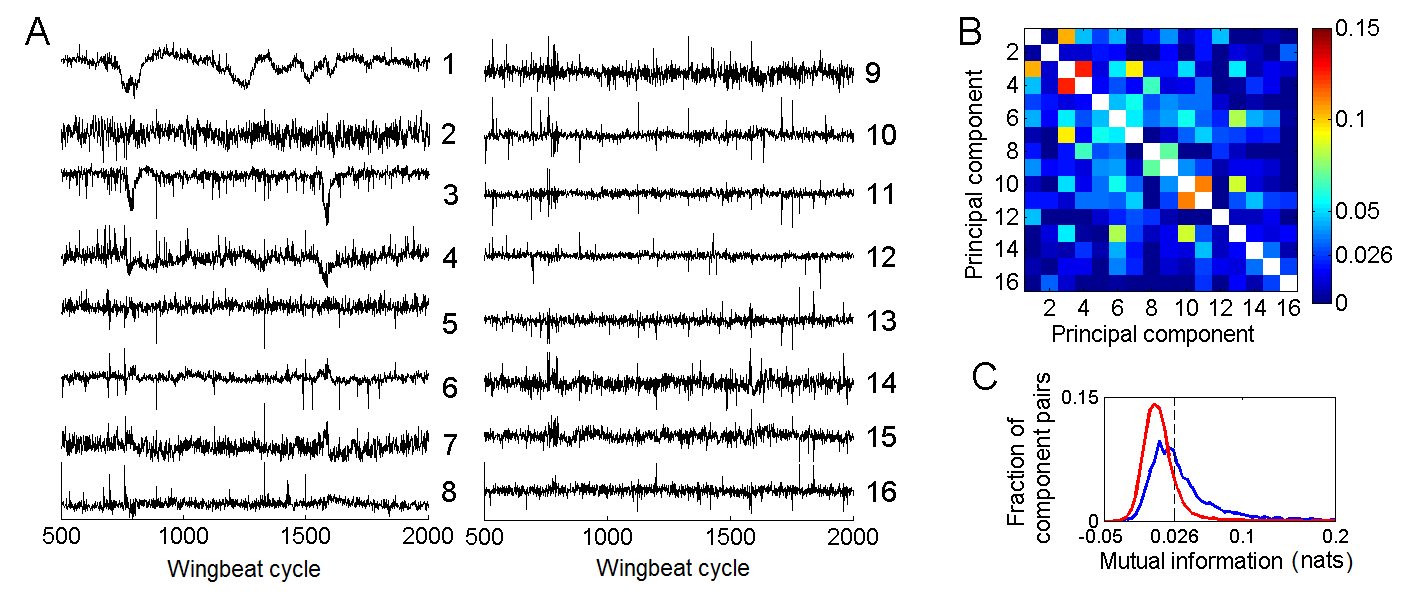

Supplement: S5 Fig — A) Time course of the principal components of signals in Fig. 6A. The sharp events dominating principal component 3 are also present in principal component 4—in contrast to the isolation of these events in only one least-dependent component (Fig. 6B). B) Dependency matrix of the principal components shown in A. The color code indicates the value of mutual information for a given pair of components. (The value on the diagonal is undefined.) Components with mutual information above 0.26 nats (threshold of significant mutual dependence) are frequent, and 5 pairs have very high mutual information (>0.9 nats). C) Distribution of pairwise mutual information, for principal components (solid blue line) and least-dependent components (solid red line) from each flight segment, over a total of 100 flight segments. The dashed black line marks the α = 0.01 confidence limit for rejecting the null hypothesis of zero mutual information. Only 56% of principal component pairs have mutual information less than 0.026 nats, in contrast to 88% of least-dependent components. (TIF) [file pone.0116813.s007.tif]
